# Age influences the temporal dynamics of microbiome and antimicrobial resistance genes among fecal bacteria in a cohort of production pigs

**DOI:** 10.1186/s42523-022-00222-8

**Published:** 2023-01-10

**Authors:** Tara N. Gaire, H. Morgan Scott, Noelle R. Noyes, Aaron C. Ericsson, Michael D. Tokach, Mariana B. Menegat, Javier Vinasco, Boyd Roenne, Tui Ray, T. G. Nagaraja, Victoriya V. Volkova

**Affiliations:** 1 Department of Diagnostic Medicine/Pathobiology, College of Veterinary Medicine, Kansas State University, Manhattan, KS 66506 USA; 2 Department of Veterinary Pathobiology, School of Veterinary Medicine and Biomedical Sciences, Texas A&M University, College Station, TX 77843 USA; 3 Department of Veterinary Population Medicine, College of Veterinary Medicine, University of Minnesota, St. Paul, MN 55108 USA; 4 Department of Veterinary Pathobiology, College of Veterinary Medicine, University of Missouri, Columbia, MO 65211 USA; 5 Department of Animal Sciences and Industry, College of Agriculture, Kansas State University, Manhattan, KS 66506 USA

## Abstract

**Background:**

The pig gastrointestinal tract hosts a diverse microbiome, which can serve to select and maintain a reservoir of antimicrobial resistance genes (ARG). Studies suggest that the types and quantities of antimicrobial resistance (AMR) in fecal bacteria change as the animal host ages, yet the temporal dynamics of AMR within communities of bacteria in pigs during a full production cycle remains largely unstudied.

**Results:**

A longitudinal study was performed to evaluate the dynamics of fecal microbiome and AMR in a cohort of pigs during a production cycle; from birth to market age. Our data showed that piglet fecal microbial communities assemble rapidly after birth and become more diverse with age. Individual piglet fecal microbiomes progressed along similar trajectories with age-specific community types/enterotypes and showed a clear shift from *E. coli*/*Shigella-*, *Fusobacteria-*, *Bacteroides-*dominant enterotypes to *Prevotella-*, *Megaspheara-*, and *Lactobacillus-*dominated enterotypes with aging. Even when the fecal microbiome was the least diverse, the richness of ARGs, quantities of AMR gene copies, and counts of AMR fecal bacteria were highest in piglets at 2 days of age; subsequently, these declined over time, likely due to age-related competitive changes in the underlying microbiome. ARGs conferring resistance to metals and multi-compound/biocides were detected predominately at the earliest sampled ages.

**Conclusions:**

The fecal microbiome and resistome—along with evaluated descriptors of phenotypic antimicrobial susceptibility of fecal bacteria—among a cohort of pigs, demonstrated opposing trajectories in diversity primarily driven by the aging of pigs.

**Supplementary Information:**

The online version contains supplementary material available at 10.1186/s42523-022-00222-8.

## Background

Antimicrobial resistance genes (ARGs) within the gut microbiome of food animals are considered to be a potential risk to animal and human health. The pig gastrointestinal tract hosts a diverse microbial community, which can serve as a reservoir for ARGs [1] that can be transferred between native and transient gut bacteria [2, 3] and further deposited into the environment via feces [4]. While the gut microbiome of pigs undergoes remarkably rapid shifts following birth [5, 6], becoming more diverse with age [7] and eventually reaching stability, the exact temporal dynamics of AMR bacteria and ARGs and any contributing factors remain largely unknown in pigs throughout their production life span.

Higher levels of AMR among fecal bacteria and diverse ARGs are reported in the gut microbiome of young animals shortly after birth, even without any recorded antimicrobial treatments [8–13]. Moreover, antimicrobial exposure has been shown to have little to no impact on the ARGs prevalence and abundance in pigs [14]; meanwhile, the abundance of ARGs in the animal gut has been shown to decrease with age, which is helpful for reducing any risks at slaughter [15, 16]. Based on data reported in our scoping review [11], we speculated that age may play the most important role in driving the temporal dynamics of AMR in pigs and motivated us to further explore the temporal dynamics of AMR during a full pig production cycle.

The establishment of gut microbial communities early in life exerts a long-term influence on pathogen colonization, immune status and the performance of growing pigs [17]. The minimizing of bacterial resistance to medically important antimicrobials among the commensal bacteria of food animals is important in order to reduce the risk of AMR transmission to humans via the food chain [18]. As ARGs can be passed horizontally between bacteria or vertically within strains in the gut microbial community, microbiome composition is an important factor to consider when evaluating AMR/ARG dynamics. Thus, understanding how the fecal microbiome changes in pigs during their entire production life span (i.e., from birth to market age) and how these changes relate to dynamics of AMR profiles in the fecal bacterial populations are of great importance.

Therefore, we evaluated the dynamics of the microbiome and AMR/ARGs among fecal bacteria in a cohort of pigs (*n* = 12 at each age-point) during a 6-month production cycle (birth to market age, at nine age-points). The fecal microbiome was characterized using data from 16S rRNA gene sequencing of microbial community DNA. We also assessed phenotypic AMR via quantification of AMR coliforms and enterococci on selective media, quantified target AMR gene copies by qPCR, and performed shotgun metagenomic sequencing of community DNA to describe AMR profiles in concert with metagenomic characterization. Our hypothesis is that the fecal microbiome composition and AMR/ARG temporal dynamics in pigs are largely a function of the host age.

## Methods

### Study design and animals

The study was performed at the Kansas State University Swine Teaching and Research Center (STRC), Kansas State University, Manhattan, Kansas, USA. The farm was managed using conventional practices common to the U.S. swine industry. The animal experiments were approved by the Institutional Animal Care and Use Committee (IACUC) of Kansas State University.

**Fig. 1:**
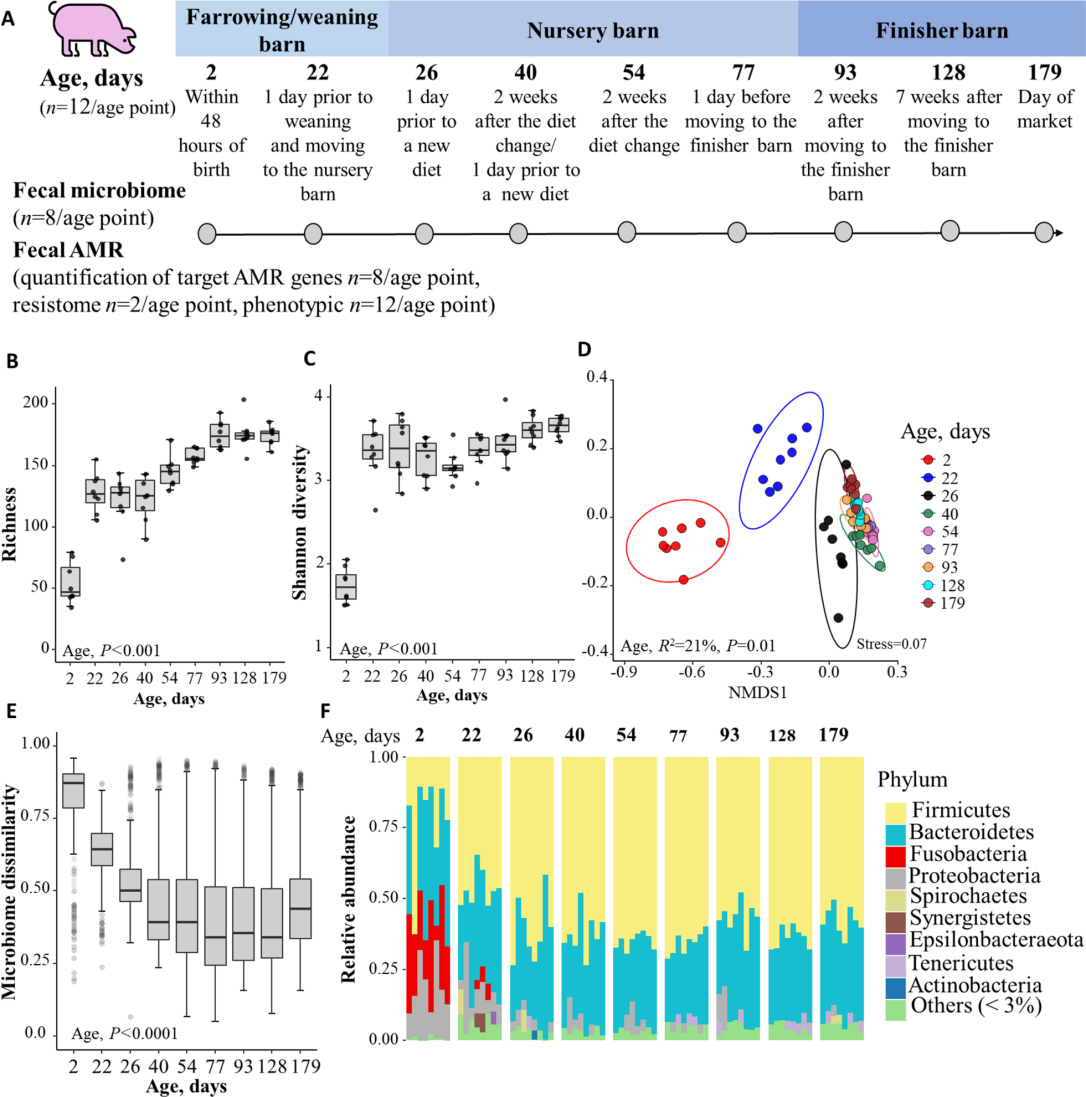
Dynamics of fecal microbiome in a cohort of pigs (2 days of age to market age). **A** The number of pigs, housing, and sampling of feces of pigs for microbiome and resistome analyses. **B**, **C** Longitudinal development of Alpha diversity (at the genus level) measured by richness and Shannon diversity. **D** Non-metric multidimensional scaling (NMDS) ordinating plot based on Bray–Curtis distances illustrate the variation in microbial community structures (i.e. beta diversity; *R*^2^ represents the amount of variability explained by age and associated *P* values based on PERMANOVA analysis), ellipses represent 95% confidence intervals for each age point. **E** Fecal microbiome community dissimilarity (Bray–Curtis dissimilarity index value) among age-points. **F** Stacked bar representing relative abundance of phyla over time; phyla with relative abundances less than 3% were grouped into “Others < 3%”

A production cohort of 16 piglets born at the STRC, balanced by sex and randomly selected from litters of eight sows, was followed from 2 days of age to the market age (~ 180 days). During the course of the study, three piglets died (one before weaning, two in late-nursery), and a fourth one lost an ear tag before moving to the finisher barn; therefore, these were removed from the study. Any samples collected from these four pigs were not analyzed. The enrolled piglets were allowed to nurse from birth to 21 days of age and then had ad libitum access to feed and water. The pigs were fed a corn and soybean meal-based diet designed to meet the nutrient requirements of each production stage. After weaning, the 12 pigs were housed in two separate pens (6 in each pen) along with pigs outside the study. None of the pigs in the two pens that housed study pigs received any antibiotics, either in feed or parenterally. A fecal sample was taken from the rectum of each pig at each of the following nine age-points: Days 2 and 22 (in the farrowing barn) and after moving to a nursery barn at 23 days of age, on Day 26 (3 days after starting a nursery diet), Day 40 (a day before another diet change), Day 54 (14 days after the diet change), Day 77 (a day before moving to a finisher barn), Day 93 (14 days after moving to the finisher barn and the introduction of a finisher diet; further, the diet was changed one more time on Day 114), Day 128 (2 weeks after the diet change), and at Day 179 (day of market) (Fig. 1A). A total of 108 fecal samples (12 pigs at nine age points) was analyzed.

Fecal samples were kept on ice after each sampling, and whole feces and fecal aliquots mixed with 50% glycerol were stored at − 80 °C until DNA extraction and quantifications of total and AMR coliforms and enterococci were performed on all samples (12 pigs/age point; total of 108 samples). A total of eight piglets (4 males and 4 females) were randomly selected using random number generation (in Microsoft Excel™, Microsoft Corporation, Redmond, WA, USA) and a total of 72 samples (*n* = 8/age point) were analyzed for community composition by 16S rRNA amplicon sequencing and for the quantifications of targeted ARGs (*tet*(A) and *bla*_CTX-M_). In addition, fecal samples from two piglets (*n* = 2 pigs (one male and one female)/at each of nine age points: total 18 fecal samples) were subjected to shotgun metagenomic sequencing to characterize the total AMR profile (i.e., the resistome). The phenotypic AMR of fecal bacteria was evaluated by quantifying total versus AMR coliforms and enterococci; these latter assays were performed on all samples (12 pigs/age point; total 108 samples).

### Fecal DNA extraction, 16S rRNA library preparation and sequencing

Total DNA was extracted from each fecal sample using the protocol published by Yui and Morrison [19], with modifications recommended by Korte et al. [20], for 16S rRNA gene sequencing. The DNA concentrations were measured via fluorometry (Qubit 2.0, Life Technologies, Carlsbad, CA) using Quant-iT broad range and high sensitivity dsDNA reagent kits (Invitrogen, Carlsbad, CA).

Extracted fecal DNA samples were processed for 16S rRNA library preparation and sequencing. The 16S rRNA amplicons (V4 region of the 16S rRNA gene) were created with universal primers (U515F/806R) [21, 22]. Briefly, 100 ng of metagenomic DNA was used, and PCR was performed in 50 µL reactions with primers, dNTPs and DNA polymerase. The PCR plate was transferred to a thermocycler for amplification and after completion of the amplification, amplicon pools (5 µL/reaction) were combined, mixed, and purified by adding Axygen Axyprep MagPCR (Axygen Corning Life Sciences, Glendale, AZ, USA) clean-up beads (50 µL beads were thoroughly mixed with 50 µL amplicons) and then incubated for 15 min at room temperature. The plate was placed on a magnetic stand for 5 min until the supernatant was cleared and then was washed with 80% ethanol. The pooled amplicon was evaluated using the Advanced Analytical Fragment Analyzer (Advanced Analytical, IA, USA) and quantified using Quant-iT HS dsDNA kits and then sequenced (2 × 250 bp paired-end reads) on the Illumina MiSeq platform (Illumina Inc., San Diego, CA).

### Processing of 16s rRNA sequencing data and assigning taxonomy

The primers were removed from reads using the Cutadapt pipeline [23]. Read pairs were rejected if one read or the other did not match a 5’primer, and an error rate of 0.1 was allowed. Quality filtering, pairing, denoising, de-replication, and determination of count of amplicon sequence variants (ASVs) was performed with the Division Amplicon Denoising Algorithm (DADA2) plugin [24] in the QIIME2 platform [25]. For quality trimming, forward and reverse reads were truncated to 150 bases based on mean quality scores ≥ 30. In order to get amplicons of the targeted length (i.e. V4 region) and to remove non-target-length sequences, bacterial 16S rRNA genes were retained only if they represented assembled sequences between 249 and 275 nucleotides in length.

Taxonomy was assigned to the sequences using the SILVA database v132 [26] of 16S rRNA sequences from bacterial species of different taxonomies using the classify-sklearn procedure. The resulting ASV table, taxonomic assignment and corresponding metadata were combined to create a *phyloseq* object using phyloseq package in R statistical software (v4.1, R Core Team 2021). The ASVs identified as other than bacteria and ASVs present in < 1% samples were discarded prior to analysis.

### Fecal resistome analysis

Fecal samples (*n* = 18) from two pigs (male and female) at each of nine age-points (i.e., Days 2, 22, 26, 40, 54, 93, 128, and 179) were subjected to shotgun metagenomic sequencing to evaluate age-related temporal dynamics of the fecal resistome in pigs. Fecal DNA was extracted from each sample using the DNeasy Power Soil Pro Kit (Qiagen, Cat No. 47016, Hilden, Germany) according to the manufacturer's instructions. Briefly, 250 mg of the fecal sample was placed in power bead tubes containing 800 µL CD1 buffer. After vortexing, bead tubes containing samples were processed on a Mini Bead-beater (Biospecproducts Cat. No. 1001, Bartlesville, OK, U.S.) at 2,200 rpm for 20 s, repeated three times with an interval of 30 s in between each bead beating cycle. Following centrifugation (15,000 × *g* for 1 min), 600 µL of supernatant was transferred into the tube, and all subsequent extraction steps were followed according to the manufacturer’s instructions, including the inhibitor removal step. DNA concentration and quality were determined by Qubit 4 Fluorometer (ThermoFisher Scientific, Cat No. Q33226, Hercules, CA, U.S.) and with Tape station genomic screen tape (Agilent Technologies, Palo Alto, CA), respectively. After DNA extraction, libraries were constructed using the Nextera DNA Flex library preparation kit (Illumina Inc., San Diego, CA, Cat. no 20018704) starting with 100 ng individual DNA samples. Paired-end sequencing (2 × 150 bp paired-end reads) was performed at the University of Minnesota Genomics Center (UMGC) in a S Prime flow cell on the NovaSeq 6000 system (Illumina Inc., San Diego, CA, USA).

To characterize the resistome composition, the sequencing reads were aligned to the AMR gene database MEGAREs v2.0 using AMRPlusPlus pipeline version 2.0 [27]. Briefly, raw sequence reads were processed, and low-quality reads and adapter contamination were removed using Trimmomatic [28]. The host-associated sequences were removed by aligning the trimmed sequences to the reference *Sus scrofa* genome using Burrows-Wheeler-Alignment (BWA) [29]. Non-host reads were aligned to the AMR gene database MEGAREs v2.0 using BWA, then converted to a SAM-formatted file using Samtools [30]; later, this was analyzed through ResistomeAnalyzer with the default set for at least 80% gene fraction (i.e., percentage of the nucleotides within the gene to which at least one read aligns). The counts of aligned sequence reads were recorded at the gene (accession), group (gene-level group for that sequence, e.g., *tet*A), mechanism (the biological mechanism of resistance, e.g., penicillin-binding protein), class (e.g., *β*-lactams, aminoglycosides) and type (drugs, metal, biocides, multi-compounds) classifications, and were normalized by cumulative sum (CSS) using a default percentile of 0.5 [31] to account for potential differences in sequence depth prior to analysis.

#### Quantification of resistance genes from fecal community DNA

In addition to metagenomic sequencing, gene copies per gram of wet feces of *tet*(A), *bla*_CTX-M_, and 16S rRNA genes were quantified in the fecal community DNA (*n* = 8/age point; total 72 samples) using quantitative real-time PCR (qPCR) as described earlier [32]. The PCR plates were set up using a dedicated robot (QIAgility™, Qiagen, Valencia, CA), and DNA was used directly as a template in qPCR reactions for quantification of the gene using the AriaMx real-time qPCR system (Agilent Technologies, La Jolla, CA). All qPCR assays were run in duplicates with two negative controls; one with a no-template control and the other DNA-free water as a template. The primer set(s) and detailed methods for standard curve generation and quantification are given in Additional file 1: Table S1. After each PCR run, data were extracted using the AriaMx ver. 1.0 software (Agilent, Santa Clara, CA). The gene copy numbers per *gram* of wet feces were determined by adjusting the dilution factor for each step of DNA extraction, and standardized (to the 16S rRNA) gene copy numbers per gram of feces were estimated.

#### Quantification of phenotypic AMR in fecal bacteria

A total of 108 samples (*n* = 12/age-points), were subjected to a culture method to quantify total and AMR coliforms and enterococci via spiral plating using an Eddy Jet 2 spiral plater (Neutech Group Inc., Farmingdale, NY, USA) as described previously [33]. The fecal samples were diluted in phosphate-buffered saline (PBS) at a 1:10 ratio before spiral plating. The 1:10 dilution was plated on MacConkey agar (Remel™, Lenexa, KS, USA) (MAC) to enumerate total coliform counts, and on MAC plates supplemented with an antimicrobial class (for each drug used for testing at clinical break‐point concentrations, see Additional file 2: Table S2) to quantity absolute and relative AMR coliform counts. Eleven antimicrobials were selected for evaluation representing nine different classes of antimicrobials: aminopenicillins (ampicillin, 32 µg/mL), aminoglycosides (gentamicin, 16 µg/mL; streptomycin, 32 µg/mL), 3rd generation cephalosporins (ceftriaxone, 4 µg/mL), fluoroquinolones (ciprofloxacin, 1 µg/mL; enrofloxacin, 0.125 µg/mL), macrolides (azithromycin, 32 µg/mL), phenicols (chloramphenicol, 32 µg/mL), quinolones (nalidixic acid, 32 µg/mL), sulfonamide/folate pathway inhibitors (sulfamethoxazole 512 µg/mL), and tetracyclines (tetracycline, 16 µg/mL).

Similarly, the 1:10 dilution was also plated on m-*Enterococcus* agar (Remel™, Lenexa, KS, USA) (ENT) to enumerate total enterococcal counts, and ENT supplemented with different antimicrobial drugs (Additional file 2: Table S2). A total of twelve antimicrobials were selected for evaluation representing nine different classes of antimicrobials: aminoglycosides (gentamicin, 500 µg/mL; streptomycin, 1024 µg/mL), fluoroquinolones (ciprofloxacin, 4 µg/mL; enrofloxacin, 4 µg/mL), lincosamides (lincomycin, 8 µg/mL), macrolides (erythromycin, 8 µg/mL; tylosin, 32 µg/mL), nitrofurans (nitrofurantoin, 128 µg/mL), penicillins (penicillin, 16 µg/mL), quinolones (nalidixic acid, 32 µg/mL) and tetracyclines (tetracycline, 16 µg/mL). The plates were incubated at 37° C for 18 h for MAC and up to 48 h for ENT. The bacterial density (CFU/g) was determined by colony counts, which were log transformed (log_10_ CFU/g) for statistical analyses. Colony counts on MAC and ENT were compared to that of media supplemented with antimicrobial compound.

### Statistical analysis

Statistical analyses were performed to evaluate age-related temporal dynamics of fecal microbiome compositions and AMR in a cohort of production pigs. All analyses were performed in R version 4.10. Figures were created using the ggplot2 package in R. We considered *P* values < 0.05 to be significant, and we used the Benjamini–Hochberg (BH) method to correct for multiple comparisons.

#### Sequencing reads

To evaluate for any potential bias due to differences in raw sequencing reads by age, sex, and housing, the numbers of raw reads generated from the V4 region of 16S rRNA sequencing from each sample were analyzed using generalized mixed models with a Poisson distribution (*glmer* function, R package). Pig identity was included as a random effect. Once a final best-fit model was selected, independent variables were evaluated using *post-hoc* pairwise comparison via the *emmeans* function implemented in R software. Model output was back transformed to original count. Similarly, the number of raw reads, filtered reads and non-host reads generated from each sample from shotgun sequencing were also summarized.

#### Microbiome analysis

##### Alpha diversity

Alpha diversity (within-sample diversity) of the microbiome as measured by taxa richness (total number of unique taxa per sample) and Shannon diversity (accounting for both richness and evenness in the abundance of taxa) were estimated for each sample. A mixed-effects linear model (R package “*lme4*”) with pig identity as the random-effect variable (accounting for the repeated measurements within each pig over time) was used to determine significant trends in alpha diversity with pig age as the predictor variable, adjusting for pig sex and housing. Type III ANOVA was used to evaluate significance of fixed effects using Anova function in R.

##### Beta diversity

Beta diversity (between sample dissimilarity in microbial composition) was computed as Bray–Curtis (BC) distances. Non-metric multidimensional scaling (NMDS) ordination plots based on the BC dissimilarity matrix (function *metamds*) using R *vegan* package [34] were used to examine how samples clustered by age and were visualized using the ggplot2 package [35]. Permutational Multivariate Analysis of Variance (PERMANOVA) was then performed on the Bray–Curtis dissimilarity matrix using the *adonis2* function in the vegan package with 999 permutations to evaluate associations among fecal microbial beta-diversity and age, sex, and housing. Individual pig identity was included as a blocking factor (“strata”) to account for repeated sampling within individuals. The multivariate dispersion for a group of samples was estimated by calculating the average distance of group members to the group centroid using the *betadisper* function in R. Then, ANOVA was performed to test if the multivariate dispersions of one or more groups (i.e., age points) were different to group centroids among samples.

To estimate the compositional divergence of the microbiome between samples, we calculated Bray–Curtis dissimilarity values for each individual pig. This value refers to how dissimilar an individual pig’s microbiome composition is from other samples, with a higher value (approaching one) indicating complete dissimilarity, and with a value close to zero indicating that two groups share the exact same number of each type of microbial taxa. We then used a linear mixed model to assess the effect of age (fixed effect) on the Bray–Curtis dissimilarity values (as the outcome variable), while including pig identity as a random intercept to account for repeated measures using the *lme* function R.

##### Identification of age-associated microbial taxa

The associations between taxonomic abundances (at phylum and genus levels) and age were determined through a multivariate modeling approach—while adjusting for sex—with the *MaAsLin2* function in R [36]. The CSS normalized microbial count matrices were the input data, and the default parameter was applied (minimal percentage relative abundance 0.01%, *P* < 0.05, *q* < 0.25). Pig identity was used as a random effect. Multiple comparisons were adjusted using the Benjamin–Hochberg FDR method.

##### Dirichlet-multinomial mixtures (DMM) community types (enterotypes)

Dirichlet multinomial mixtures (DMM) [38] were used to determine the enterotypes (i.e. microbial community types) of microbial community, as determined by 16S rRNA sequencing. The CSS normalized microbial count matrices (after removing those ASVs present in less than 5% samples) were used as input in the DMM model (R package DirichletMultinomial). The optimal number of clusters was determined based on the lowest Laplace approximation. The proportion of samples occupying a DMM cluster at each age-point was determined, and changes in enterotypes over time during the production stages were visualized [39].

##### Quantities of the 16S rRNA gene copies

Additionally, quantities of the 16S rRNA gene copies (log_10_ transformed 16S rRNA gene copies as measured by qPCR) were evaluated as a proxy of overall bacterial load [37] using a linear mixed modeling approach (including age and sex of piglets as fixed effects and pig identity as a random effect).

#### Phenotypic AMR and resistome analysis

##### Phenotypic AMR

Changes in the antimicrobial-resistant coliform and enterococci (expressed as log_10_ CFU/g feces) counts were analyzed using zero-inflated Gaussian mixed models (*lme.zig* function, NBZIMM R package) [40] on data from pigs that had at least one positive count for a given antimicrobial. This function allowed analysis of zero-inflated count responses with multilevel data structures. Pig identity as a random intercept was included to adequately control for repeated measures. Because coliforms and enterococci did not grow in the presence of fluoroquinolones (ciprofloxacin, 4 µg/mL; enrofloxacin, 4 µg/mL), and coliforms alone did not grow in the presence of quinolones (nalidixic acid, 32 µg/mL), they were excluded from the dataset and analyses.

The changes in total coliforms and enterococci as measured via the log_10_ CFU/g counts (i.e., without antimicrobials) were analyzed using generalized linear mixed models (“*lme4*” package in R software) [41]. The pig identity was used as a random effect to account for the lack of independence among the serial fecal samples. Estimated marginal means were extracted using *lsmeans* function in R*.*

##### Resistome analysis

Similarly, for the resistome analysis, filtered (i.e., after filtering those required single nucleotide polymorphisms confirmations) and normalized resistome count matrices were used to summarize resistome composition by the age of pigs. The richness of ARGs was defined as the observed number of unique ARGs (AMR gene group) present in a sample and was estimated separately for drugs, metal, biocides, and multi-compound resistome types, as well for the total of all of these (i.e., combined drugs, metal, biocides, and multi-compounds), using the *alpha* function in the *microbiome* package in R. The fecal resistome composition at the ARG level, and mechanisms, were visualized using the NMDS ordination plot based on Bray–Curtis distance as described above. Associations between age and resistome composition were analyzed similarly to the 16S data using PERMANOVA, with the exception that the age was categorized into three groups (i.e., 2–22, 26–77, and 93–179 days of age), representing when pigs were housed in farrowing-weaning, nursery, or finisher barns, respectively. Changes in ARG richness and abundance of ARGs (i.e., differential abundance testing) between the ages of piglets were not formally compared (statistically) due to the small sample sizes.

A *post-hoc* co-occurrence network analysis [42] was performed between ARG types (i.e., ARGs conferring resistance to drugs, metal, multi-compounds and biocides). ARGs present in at least 5 (of 18) piglet’s fecal samples were included in the co-occurrence correlation analysis. A correlation matrix was constructed by calculating all possible pairwise Spearman’s rank correlations (*ρ*) between resistance genes (ARGs) on CSS normalized count metrices. Correlation coefficients (*ρ*) that were > 0.6 and significant after Benjamini–Hochberg multiple correction (*P adjusted* < 0.01) were included in co-occurrence networks and visualized using Gephi v.0.9.2 [43].

##### Quantification of targeted ARGs

For qPCR data, copy numbers for each of *tet*(A) and *bla*_CTX-M_ genes were standardized to the 16S rRNA gene copies representing the total bacterial population in each gram of feces. All standardized gene quantities, as well as total 16S rRNA gene copies, were logarithmically transformed to base 10 for use as an outcome variable in linear mixed models. The models accounted for animal-level dependencies as random effects. Missing values often were recorded for *bla*_CTX-M_ genes, mostly for samples collected on days 77 of age, indicating they were either below the detection limit, below the threshold limit determined by the test, or else truly absent. Many of these observations were expected to comprise more than true zero. Thus, estimates of missing values were imputed using a multiple imputation method [44] in STATA MP 16.1 (STATA Corp., College Station, TX) and multiple regression methods were employed for the imputation process. Pig identity was included in the imputation model and twenty imputations were performed to address the variability arising from the imputation process [32].^.^ The effects of age on *bla*_CTX-M_ gene quantities were determined using a multilevel mixed linear model (*lme* function in R) on the expanded dataset following imputation. In addition, results of the main regression model before imputation were also performed and compared to those including imputed values.

## Results

### Fecal microbiome changes over time

To assess the temporal dynamics of the fecal microbiome from birth to market age in a cohort of piglets, fecal samples (*n* = 8/age-point) were subjected to ribosomal RNA gene sequencing analysis (Fig. 1A). The 16S rRNA gene (V4 region) sequencing generated a total of 9.4 million (range: 86–168 K; average 130.8 K; median: 129 K) paired-end sequencing reads across all 72 samples. The average counts of raw reads varied across the age-points (generalized linear mixed model/glmer *P* < 0.001), with samples collected on Day 2 of age having, on average, 24 K higher read counts than at Day 22 of age (*P* = 0.001); meanwhile, Day 22 of age had, on average, 30 K and 27 K fewer reads than at 40 and 93 days of age, respectively (both *P* < 0.001). Similarly, samples collected on Day 40 of age had, on average, 19 K, 21 K and 22 K higher reads than at 77, 128 and 179 days of age, respectively (all *P* < 0.01) (Additional file 3: Table S3). Additionally, samples collected on Day 93 had, on average, 18 K and 19 K higher read counts than on Days 128 and 179 of age (*P* = 0.045, *P* = 0.039). Importantly, average reads were similar when comparing male and female pigs (*P* = 0.773) and among housing types (farrowing/weaning barn, nursery barn, and finisher barn; *P* = 0.427). After DADA2 filtering, a total of 8.6 million reads remained across the samples, and a subsequent total of 6.1 million sequencing reads remained after removing the chimera reads (Additional file 3: Table S3). CSS normalization microbial count matrices were generated to avoid the potential for sequencing depth bias. After removing 30 ASVs that were classified as ‘*other than bacteria*’, 7,048 ASVs (mean ± SD per sample: 502 ± 225, range: 74–870) were included in downstream analyses. Most ASVs could be taxonomically assigned to the phylum (100%), class (93%), and order (92%), with assignments decreasing substantially at the family (79%) and genus (67%) levels.

Our results showed that the fecal bacterial community in piglets was established rapidly (within 48 h) after birth and that fecal microbial diversity increased with age. The alpha diversity (within-sample diversity) of bacterial genera as measured by both richness and the Shannon diversity index significantly increased (LMM, *P* < 0.001 for both models) (Fig. 1B, C) as the pigs grew older and reached a relatively stable apex during the finishing stage from 128 until 179 days of age. This increasing trend of richness and diversity of the microbial community was also observed at the lowest taxonomic level (ASVs) (Additional file 4: Fig. S1 A–B). Furthermore, age was one of the strongest predictors of the difference in the overall bacterial community composition at nearly all taxonomic levels (Bray–Curtis, PERMANOVA, *R*^2^ = 16–41%, *P* < 0.001) (Fig. 1D, Additional file 4. Fig. S1 C–F) and the samples clustered tightly by the age of pigs. Sex (*R*^2^ =  < 1%, *P* = 0.001) of pigs and housing (*R*^2^ = 13%, *P* = 0.001) contributed lesser variation to bacterial community composition than age.

In addition, the Bray–Curtis (BC) dissimilarity value, which is a measure of the stability of the bacterial community, steadily decreased in the first 77 days of age and reached its nadir between 93 and 128 days of age; albeit, with a slight rise in dissimilarity at 179 days of age. Using a linear mixed model, we found that pig fecal bacterial community composition had a significantly higher dissimilarity (i.e., higher BC dissimilarity values) (LMM ANOVA, *P* < 0.0001) at Day 2 sampling (estimated mean: 0.98, 95%CI: 0.79–0.82) compared to the rest of the age-points (Fig. 1E). In addition, variation of fecal microbiome composition among individual animals was also greatest at the earliest age, with a decrease and then leveling at approximately 2 months of age (beta-dispersion, ANOVA, *P* < 0.001, Additional file 5: Fig. S2).

Across all samples, over 50% and 30% of the fecal microbiome were represented by bacteria belonging to phyla *Firmicutes* and *Bacteroidetes,* respectively (Fig. 1F). Among 2-day-old piglets, *Bacteroidetes* was the most abundant phylum, accounting for 34% of the total, followed by *Fusobacteria* (~ 24%), *Firmicutes* (~ 23%), and *Proteobacteria* (~19%). Using MaAsLin2 multivariate time-series analysis, we found the relative abundances of four phyla, *Fusobacteria*, *Proteobacteria*, *Actinobacteria*, *Bacteroidetes,* were negatively associated with days of age (Benjamini–Hochberg false discovery rate (FDR) adjusted *P* < 0.05). In contrast, the relative abundances of phyla *Tenericutes*, *Cyanobacteria*, *Spirochetes*, *Kiritimatiellaeota*, and *Patescibacteria* increased significantly with age (Benjamini–Hochberg false discovery rate (FDR) adjusted *P* < 0.001) (Additional file 6: Fig. S3A). The relative abundance of *Firmicutes* began to increase the day prior to weaning, but was not significantly associated with age between 2 and 77 days of age; thereafter, it started to decline up to Day 179, or market age (BH adjusted *P* < 0.01).

Additionally, we identified 24 genera that were negatively associated with age and a further 66 genera that were positively associated with the age of pigs (BH. adjusted *P* < 0.01) (Additional file 7: Table S4). The relative abundances of several genera, such as *Escherichia/Shigella*, *Enterococcus*, *Clostridioides*, *Fusobacterium*, *Clostridium *sensu* strictu*, *Ruminococcus gnavus group*, *[Eubacterium] fissicatena group*, *Actinobacillus, Veillonella*, *Hungatella*, *Eisenbergiella* continuously decreased as the pigs aged (BH adjusted *P* < 0.001). In contrast, the relative abundances of *Alloprevotella*, *Blautia*, *Christensenellaceae R-7 group*, *Lachnospiraceae FCS020 group*, *Mogibacterium, Ruminococcaceae UCG-014,* etc.*,* (complete list is included in Additional file 7: Table S4) increased throughout the production cycle (from day 2 of age until day 179).

We further evaluated changes in log_10_ 16S rRNA gene copies per gram wet feces as a measure of absolute abundance by age of piglets. At day 2 of age, mean log_10_ 16S rRNA gene copies per gram ± S.E. was 10.6 ± 0.17, and did not change significantly over time (*P* = 0.08) or vary between males and females (*P* = 0.315) (Additional file 6: Fig. S3C), suggesting that absolute abundance of bacterial community per gram feces remained relatively similar throughout the study period.

### Age-specific bacterial enterotypes

**Fig. 2:**
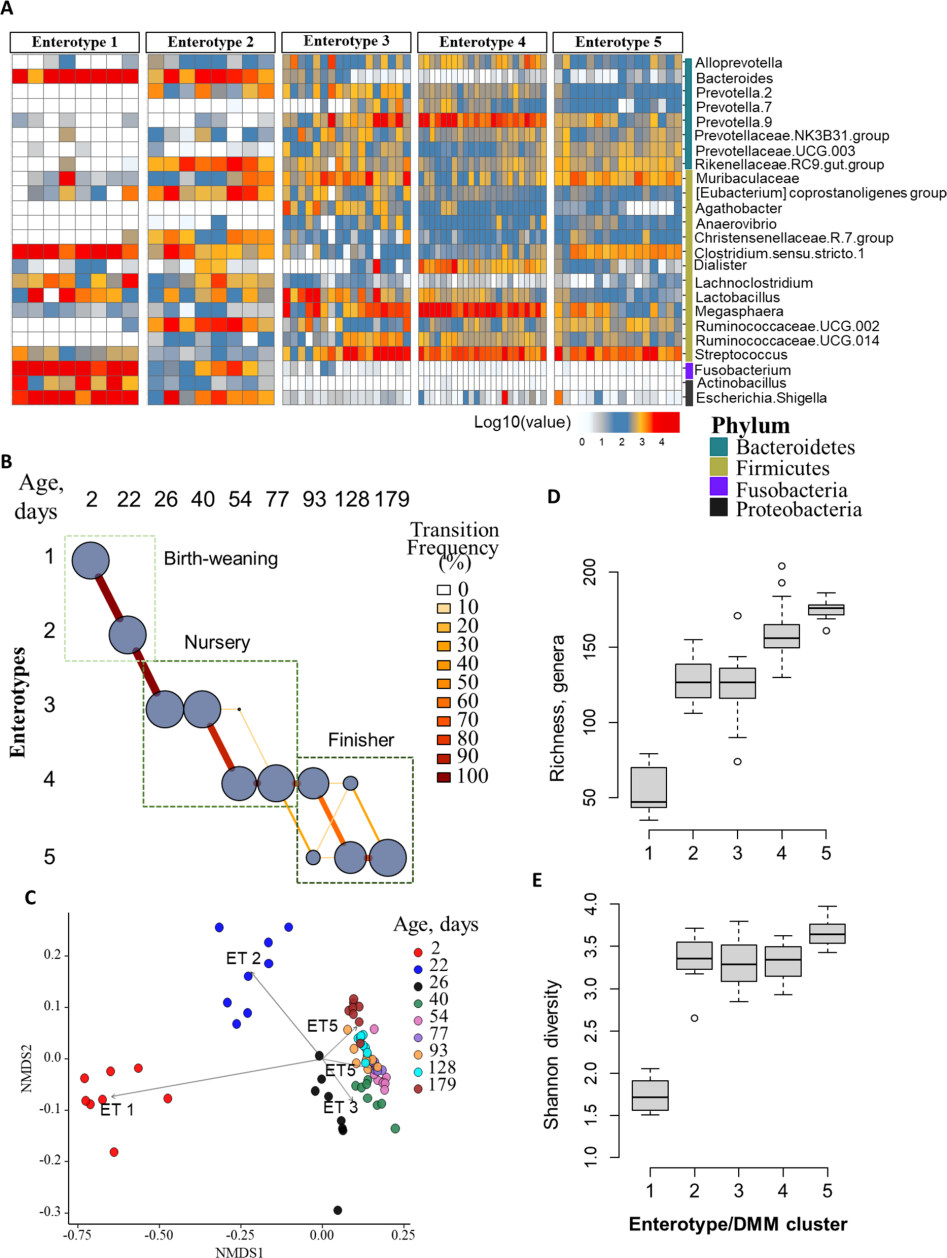
Fecal microbiome development through age-related bacterial community types (i.e., Enterotypes). **A** Heat map of the top 25 most abundant genera (rows) per Dirichlet multinomial mixtures identified DMM cluster/enterotype. **B** Transition model illustrating progression of samples through enterotypes (y-axis) per each age-point (top-x axis) among a cohort of pigs. Color intensity and line thickness are scaled by the number of piglets in the transition. **C** NMDS plot showing overall microbial composition with enterotypes (ET 1–5 represent Enterotypes). **D**, **E** Box plots showing the alpha diversity indices (richness and Shannon’s diversity) per each Enterotypes/DMM cluster

We next sought to determine microbiome progression with age in pigs by determining enterotypes based on DMM modeling with 16S rRNA gene sequencing data. The appropriate numbers of clusters were determined based on the lowest Laplace approximation score. All (*n* = 8/at each of nine age-points, total *n* = 72) 16S rRNA gene sequencing data at the genus level formed five clusters or enterotypes (Additional file 8: Fig. S4). A DMM transition model [39] was applied to evaluate the progression of samples through each DMM cluster/enterotypes with age. At 2 days of age, samples from all pigs occupied Enterotype 1 in which *Bacteroides, Fusobacterium,* and other facultative anaerobic genera, such as *Escherichia/Shigella*, *Actinobacillus* were most dominant (Fig. 2A, B). Interestingly, *Enterobacteriaceae* represented ~ 16% of the fecal microbiome in the 2-day-old piglets, but the relative abundance decreased over time to less than 1% (Additional file 6: Fig. S3 B) at market age. However, at 22 days of age, a day prior to weaning, 100% of pigs transitioned from Enterotype 1 to 2. Enterotype 2 was different from 1 with substantial depletion in the relative abundances of genera belonging to phyla *Proteobacteria* and *Fusobacteria*, and with an increase in the relative abundances of taxa belonging to the phyla *Firmicutes* (e.g., *Ruminococcus*, *Eubacterium*, *Lactobacillus*) and *Prevotella*. Shortly after weaning, at 26 and 40 days of age in the nursery phase, the piglet’s fecal microbiome transitioned to Enterotype 3, in which *Lactobacillus*, *Megasphaera* and *Streptococcus* genera were most dominant*.* As pigs aged, their microbiome transitioned into Enterotype 4 at 54, 77 (nursery stage), and 93 days of age (i.e., 2 weeks after moving into the finisher facility) and into Enterotype 5 at 128 and 179 days of age of the finishing pigs. Enterotype 4 was dominated by *Megasphaera*, *Prevotella*, and *Streptococcus*, while Enterotype 5 was dominated by *Muribaculacae*, *Ruminococcocae*, *Clostridium*, and *Streptococcus* (Fig. 2A, B).

These results were further supported by the clustering pattern of samples based on their overall microbiome profile (NMDS DMM) (Fig. 2C). The alpha diversity indices of these enterotypes varied, with Enterotype 1 of neonates being the least diverse and Enterotypes 4–5 the most dominant during the finisher phase with highest alpha diversity indices (Fig. 2D, E).

In summary, our results showed that the fecal microbiome of a cohort of pigs increased in diversity over time, with multiple transitions to different enterotypes as the pigs aged and finally reached relative stability at approximately 128 days of age of their production life span.

### Age specific resistome and targeted ARGs

Shotgun sequencing of 18 samples (*n* = 2 pigs/age point, nine age points, Fig. 1A) yielded a total of 258.4 million paired-end reads with an average of 14.4 million (range 10.6–17.5 M). The number of raw paired-end reads remained similar across all age points, indicating similarly sequencing reads across samples (Additional file 9: Fig. S5). Only 1.1% of reads were removed due to low quality. The proportion of host (*Sus scrofa)* DNA in the samples was variable, with an average of 35% (range: 21–52%). The remaining 149.2 million non-host reads were then used to aligned to the resistome. Across all samples, ~ 0.2% of non-host reads aligned to ARGs within the MEGARes database.

**Fig. 3:**
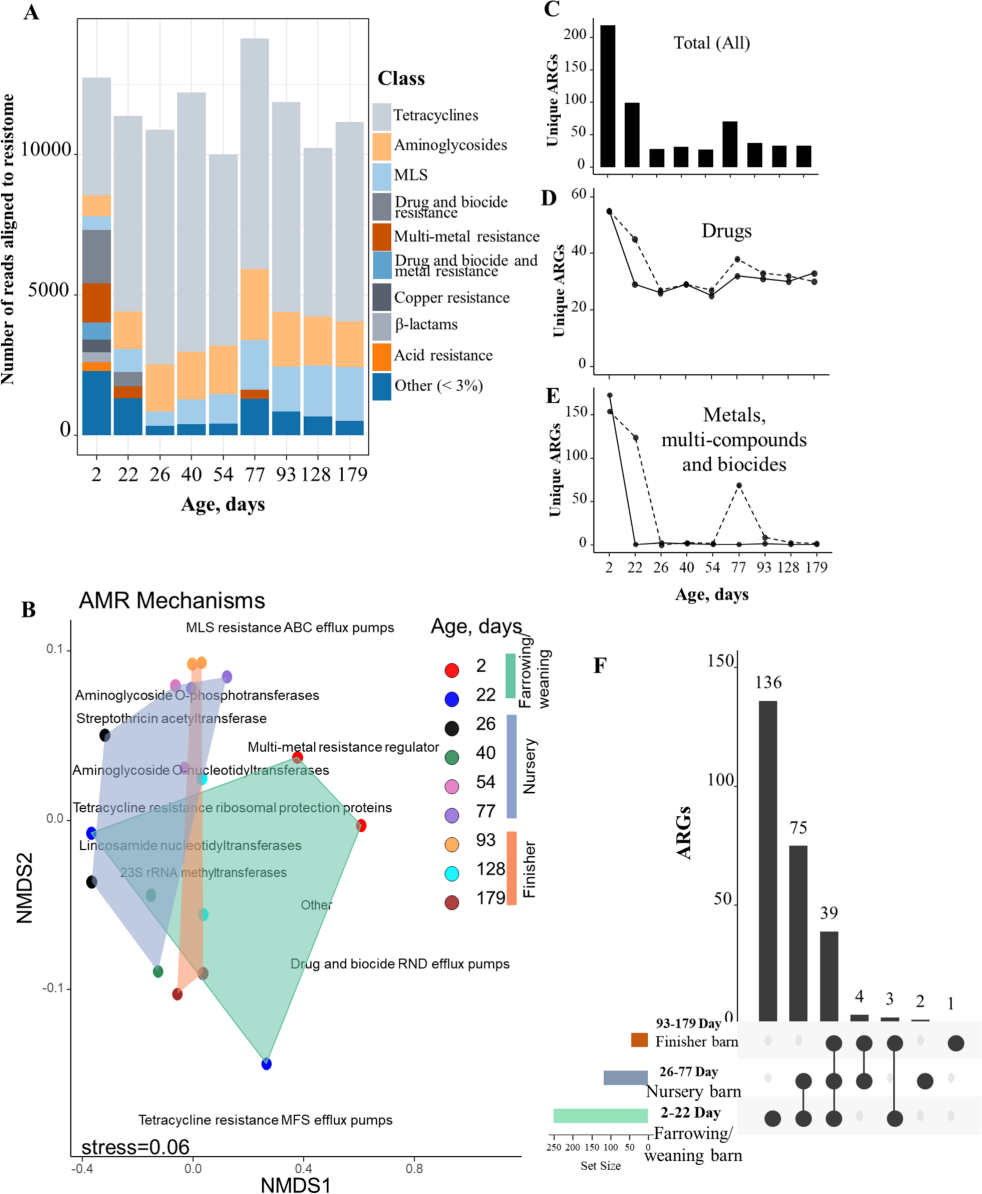
The fecal resistome composition of pigs. **A** Abundance of antimicrobial resistance genes (ARGs, i.e., number of reads aligned to ARGs within the MEGARes v2.0 database) by antimicrobial class over time. The ARGs counts were normalized using the CSS method. **B** Non-metric multidimensional scaling (NMDS) based on normalized count of ARG by AMR mechanism and biplot of AMR mechanism (annotated text represented AMR mechanism) showing overall changes in resistome composition with age. Polygons were applied to age groupings (Days 2–22, Days 26–77 and Days 93–179 of age. **C** Number of unique ARGs identified at each age-point. **D**, **E** Number of unique ARGs conferring resistance to drugs only, and ARGs conferring resistance to other categories (metal, multi-compounds, and biocides), respectively.). **F** Number of ARGs common within each age-group

A total of 260 unique ARGs, which represented 72 AMR mechanisms and 29 AMR classes, was detected across all samples; of these, the most abundant resistance type was drug resistance (90% of all ARGs), followed by metal (4.7%), and multi-compound (3.3%) resistance. Overall, tetracycline resistance genes comprised the largest proportion of the resistome (~ 60% of total ARGs), followed by aminoglycoside (14.5%), macrolide-lincosamide-streptogramin (MLS; ~ 10.5%), multi-metal resistance (2.6%), and other drug and biocides resistance genes (2.3%) (Fig. 3A). The relative abundance of ARGs conferring resistance to multi-drug, multi-metal, drug and biocides, multi-biocides, bacitracin, beta-lactams, as well as several other drug classes decreased with age, particularly during the post-weaning period (Fig. 3A). However, some of the drug classes, such as aminoglycosides, MLS, and tetracyclines, remained relatively stable throughout the ~ 180-day production cycle. Of the 260 unique ARGs detected, a total of 77, 97, 61, and 25 unique ARGs conferred resistance to drugs, metals, multi-compounds, and biocides, respectively. Overall, *tet*(w), *tet*(O), *tet*(40), *ant6*, *tet*(Q), *tet*(L), *tet*(44), *aph2*(DPRIME), *lsa*, *lmu*C, sat, and *erm(*B) were the most abundant ARGs across all the samples.

Overall, the fecal resistome significantly differed (PERMANOVA, R^2^ =  ~ 50%, *P* = 0.001, Fig. 3B, Additional file 10: Fig. S6) by pig age group (i.e., 2–22, 26–77 and 93–179 days of age). The results indicated that 2-day-old piglets’ resistome was different from older groups (Fig. 3B) and also carried the highest number of unique ARGs in their fecal samples, representing 72 AMR mechanisms. Tetracycline resistance ribosomal protection proteins, drug and biocide resistance nodulation cell division (RND) efflux pumps, multi-metal resistance proteins, drug and biocide major facilitator superfamily (MFS) efflux pumps, and drug and biocide and metal RND efflux pumps were the most abundant AMR mechanisms detected in 2-day-old piglets and drove the majority of separation from the other two age groups. Interestingly, 22%, 21%, and 7% of total normalized ARG abundances observed in the 2-day-old piglet samples were attributed to metals, multi-compounds, biocides, respectively, with the remaining 49% associated with drug resistance.

The richness of ARGs sharply decreased from 2 days of age (~ 225 unique ARGs) to 99 ARGs at 22-days of age, and further declined until the day of market (~ 35 unique ARGs) (Fig. 3C). Notably, the temporal dynamic of ARGs that conferred resistance to metals, multi-compounds and biocides rapidly diminishing postweaning, while the number of ARGs that conferred drug resistance remained relatively stable following postweaning (Fig. 3D, E). In addition, a total of 61 unique ARGs were only detected in samples from 2 days of age (data not shown), further supporting the idea that newborn piglets harbor more unique resistant genes within their fecal microbiome than older pigs. Furthermore, a total of 136 ARGs detected in samples from 2 and 22 days of age were not detected in any other samples collected from older pigs at days 26 to day 179 (Fig. 3F). In addition to the changes in the fecal resistome composition, the total resistome counts (i.e., number of sequence reads aligned to the resistome) also changed as the pigs aged (Fig. 3A, Additional file 9: Fig. S5B), while the total number of raw reads remained largely similar.

### Correlations among ARG types

The interrelationship among ARG types (i.e., ARGs that conferred resistance to drugs, metals, multi-compound, and biocides) was assessed using co-occurrence network analysis based on ρ > 0.6, and BH-adjusted *P* < 0.01 correlations. Network analysis showed that ARGs that conferred resistance to drug, metal, multi-compound and biocides were correlated in a cohort of production pigs, with 34 ARGs (nodes) and 67 edges. Several ARGs that encode resistance to drugs (e.g., *aph3*(DPRIME), ANT9, *lnu*B, *lsa*E, *tet*(A)), metals (e.g., *zin*T, *ygj*H), biocides (e.g., BPB), and multi-compound resistance genes (e.g., *CRP*, *sox*S) were connected (Additional file 11: Fig. S7).

#### Quantities of ***tet***(A) and ***bla***_CTX-M_ genes copies in feces

**Fig. 4:**
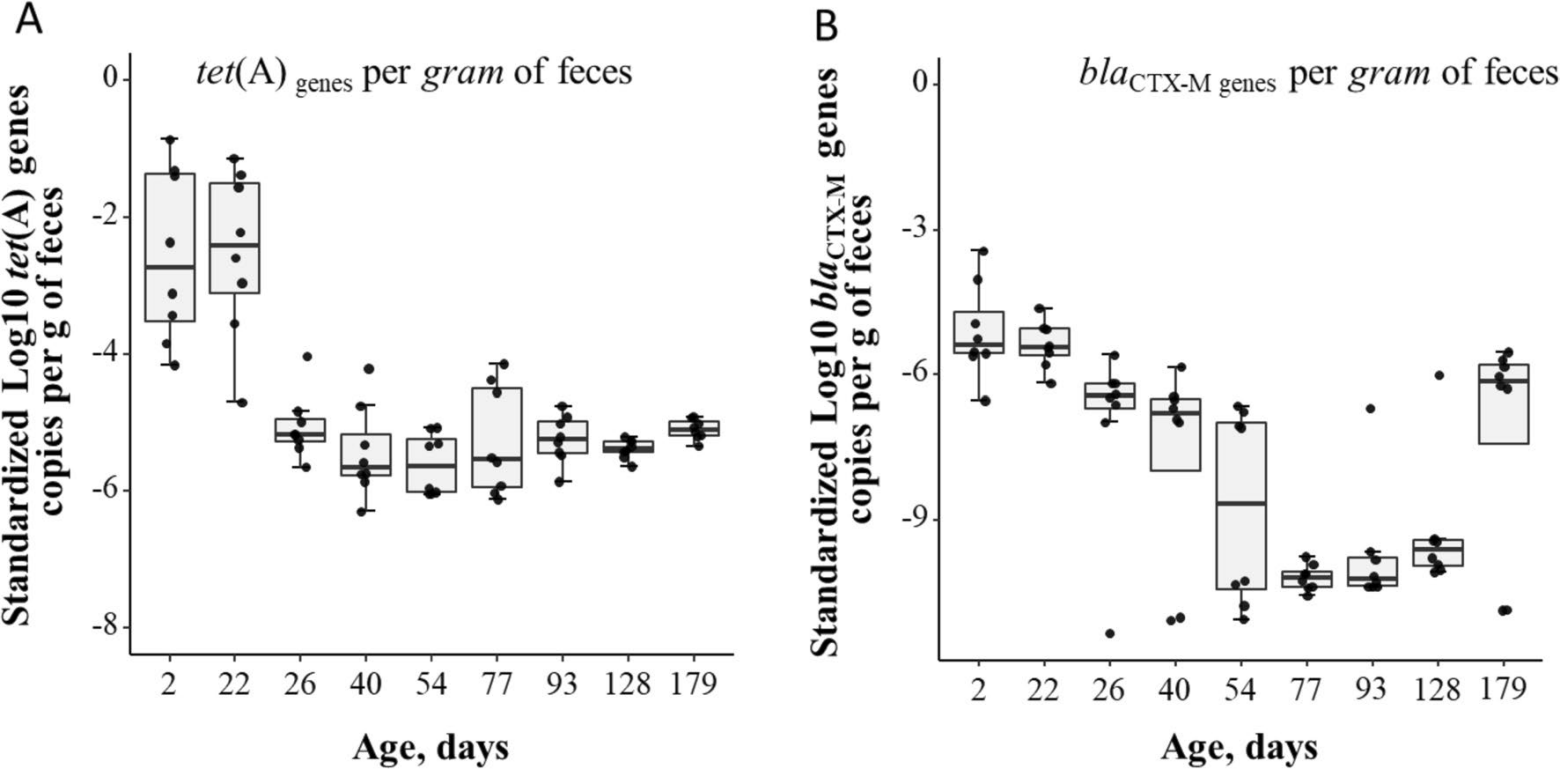
Standardized mean log_10_ copies of resistance genes. **A** tetracycline *tet*(A), and **B** 3rd generation cephalosporin *bla*_CTX-M_ genes per gram of wet fecal samples from pigs at different ages. Non-zero values (log10 + 1) were assigned to *bla*_CTX-M_ genes for graphical purposes

Standardized log_10_ copies per gram of feces for each of the two genes are presented in Fig. 4. When we evaluated 16S rRNA gene copies measured by qPCR as absolute bacterial abundance, we found mean log_10_ 16S rRNA gene copies were relatively similar across all age points (*P* = 0.08) and between male and female pigs (*P* = 0.315) (Additional file 6: Fig. S3C). Mean log_10_ copies of standardized *tet*(A) decreased significantly (*P* < 0.0001) with age; starting at – 2.6 copies of gene per 16S rRNA gene at 2 days of age, then sharply declining to – 5.1 copies of gene per 16S rRNA gene from day 22 to 26 days of age and then remaining similar until 179 days of age (Fig. 4A).

In the study, approximately 40% of samples were negative for the *bla*_CTX-M_ gene. Missing observations were mainly in samples taken from pigs at 77, 93 and 128 days of age. Non-zero values were initially assigned for graphical purposes (Fig. 4B). The lower and upper limits of detected log_10_ copies of *bla*_CTX-M_ were 3 log_10_ and 6 log_10_ gene copies per gram of wet feces, respectively. Multiple imputation procedures were performed and found to be efficient in imputing values for the missing observations in the dataset. Twenty multiple imputations (Additional file 12: Fig. S8A) were performed and used in subsequent regression analyses. The effects of age on *bla*_CTX-M_ genes quantities were determined using a multi-level mixed linear model on the dataset following imputation. Overall, mean standardized *bla*_CTX-M_ gene quantities (following imputation) varied by age of pigs (*P* = 0.07), with samples from pigs 2 days of age harboring significantly (*P* < 0.05) higher gene quantities compared to those at 26, 40, 54 and 179 days of age (Additional file 12: Fig. S8B). In addition, analysis of the non-imputed dataset (after excluding missing observations) showed that standardized *bla*_CTX-M_ gene quantities were higher at the earliest age point and declined with age until day 77, later increasing to 179 days of age. The linear mixed model analysis results showed that mean standardized *bla*_CTX-M_ gene quantities significantly decreased with age (*P* < 0.001), with samples from 2 and 22 days of age harboring higher gene quantities than at 26, 40, and 54 days of age.

### Phenotypic AMR indicator fecal bacteria were highest at the earliest age in piglets

**Fig. 5:**
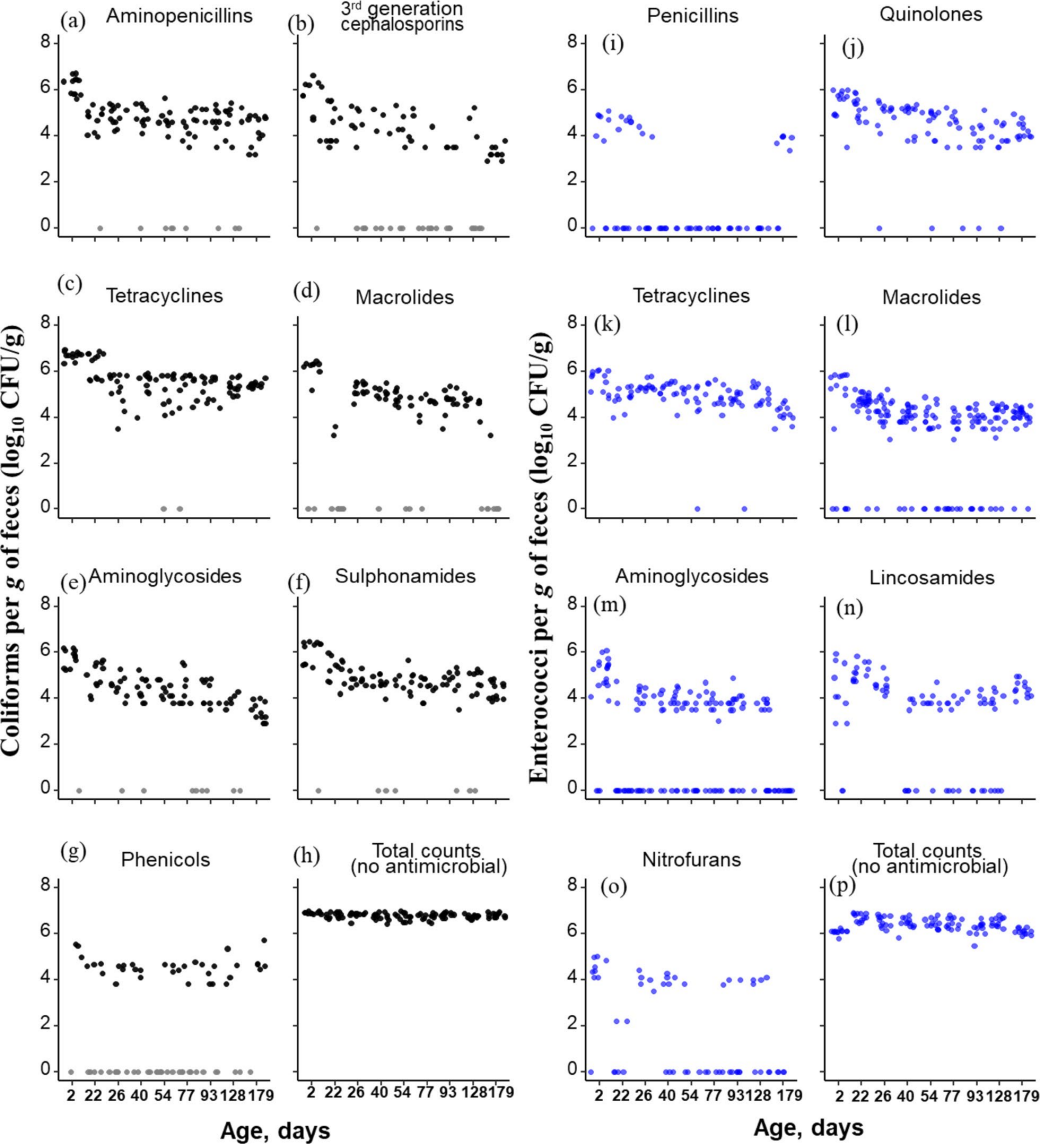
Change in viable counts (log_10_ CFU per g of wet feces) of fecal coliform (**a**–**h**) and enterococci (**i**–**p**) in the presence of clinical break‐point concentrations of each antimicrobial in relation to pig age

Sixty-three percent and 58% of all samples (*n* = 108) carried coliforms and enterococci, respectively, that were resistant to at least one of the antimicrobials tested. Overall, the counts of AMR coliforms and enterococci were detected as highest at the earliest age and gradually declined with increasing age of pigs. On average, across all ages, pigs carried 6.8 ± 0.1 log_10_ CFU/g of coliform bacteria (i.e., growth without antimicrobials) and 6.4 ± 0.3 log10 CFU/g of enterococci. Total coliform counts remained relatively stable over time and did not vary with age (*P* = 0.155); further, these were not associated with the sex of the pigs (*P* = 0.23). However, total enterococcal counts did vary with age (LMM, *P* = 0.001), with samples from Day 2 of age being of relatively lower counts compared to 22 and 77 days of age; that said, the difference in CFUs was low (~ − 0.52 log_10_ CFU/g), and the proportion of samples yielding total enterococci remained unchanged. Changes in viable count (log_10_ CFU/g) profiles of AMR coliforms and enterococci and their totals (i.e., without antimicrobials) in the cohort of pigs are shown in Fig. 5.

Increasing age was associated with a decline in the counts of both coliforms and enterococci that were resistant to one or more antimicrobials. Across all pigs and ages, aminopenicillin-resistant coliforms were detected in 89%, third-generation cephalosporin—(i.e., ceftriaxone) resistant coliforms in 56%, tetracycline-resistant coliforms in 96%, aminoglycoside-resistant coliforms in 41%, macrolide-resistant coliforms in 67%, sulfonamide-resistant coliforms in 88% and phenicol resistant coliforms in 33% of fecal samples. Two-day-old piglets exhibited the highest levels of AMR coliforms (log_10_ CFU/g) in their feces. On average, two-day-old piglets carried 6.2 ± 0.4 log_10_ CFU/g aminopenicillin-resistant coliform bacteria, 4.7 ± 2.3 log_10_ CFU/g cephalosporin-resistant, 6.7 ± 0.2 log_10_ CFU/g tetracycline resistant, 4.6 ± 2.8 log_10_ CFU/g macrolides resistant, 5.3 ± 1.7 log_10_ CFU/g aminoglycoside-resistant, 5.5 ± 1.8 log_10_ CFU/g sulfonamide-resistant and 1.3 ± 2.4 log_10_ CFU/g phenicol-resistant coliform bacteria; however, except for the phenicol counts, average log_10_ CFU/g were significantly changed (all *P* < 0.05) over time, declining with pig age (Fig. 5A–H).

Across all samples, penicillin-resistant enterococci were detected in 21% of samples, quinolone—(nalidixic acid) resistant enterococci in 82%, tetracycline-resistant enterococci in 97%, aminoglycoside-resistant enterococci in 39%, macrolide-resistant enterococci in 72%, lincosamide-resistant enterococci in 70% and nitrofuran resistant enterococci in 25% of samples. Similar to coliforms, higher counts of AMR enterococci were observed in the youngest ages of pigs. For instance, at 2 days of age, piglets carried 2.7 ± 3.9 log_10_ CFU/g penicillin-resistant enterococci; thereafter, this gradually decreased with age. Similarly, at 2 days of age, on average, piglets carried 5.4 ± 0.73 log_10_ CFU/g quinolone—(nalidixic acid) resistant enterococci, 5.6 ± 0.45 log_10_ CFU/g tetracycline-resistant, 2.4 ± 2.7 log_10_ CFU/g of macrolide-resistant, 3.8 ± 2.3 log10 CFU/g aminoglycoside-resistant, 3.3 ± 2.2 log_10_ CFU/g aminoglycoside-resistant, and 3.0 ± 2.2 log_10_ CFU/g nitrofuran-resistant enterococci in feces, all of which declined with age (all *P* < 0.001) (Fig. 5I–P). There was no association between AMR coliform or enterococci counts (log_10_ CFU/g) and the sex of pigs (all tested antimicrobials, *P* > 0.05).

## Discussion

This study focused on the temporal dynamics of the microbiome and AMR/ARGs in the feces of a cohort of pigs from birth to marketing for slaughter. We demonstrated that the fecal microbiome of pigs undergoes consistent change from birth to market age, with a concurrent reduction in both unique ARGs and viable counts of fecal AMR indicator bacteria resistant to one or more drug classes. The detection of high levels of ARGs and AMR gene copies in the gut microbiome within 48 h post birth in piglets, and their subsequent decline, suggests that initial establishment of ARGs and AMR bacterial populations is independent of antimicrobial exposure, and that their declining trajectories during the production process is primarily driven by the age of pigs.

### Fecal microbiome variations are associated with age

Our results are similar to those reported in previous studies [14, 45–48] concerning the dynamic changes among fecal bacterial populations in pigs at various production stages. As expected, age was the strongest predictor of the diversity and composition of the fecal microbiome. We observed individual pig fecal microbiomes becoming more diverse with age in the first 3 months of production life, then reaching relative stability during the finishing period (i.e., from 128 to 179 days of age). Our results are also consistent with human infant gut microbiome studies [49–52], which demonstrated age-related changes in the gut microbiome. The pig fecal microbiome is more compositionally homogeneous with respect to age and reached relative stability at approximately 3 months of age, suggesting the development maturation and stability of the gut microbiome in growing pigs is a non-random process and is primarily age driven. Of course, the consistency of diet in pigs reared in a single production facility may be more alike than across those households included in human microbiome studies of infants. Similar trends showing an increase in microbiome diversity with age were also reported in the human adult population [53], further indicating age as a dominant factor that can lead to significant variation in the gut microbiome.

We observed longitudinal age-specific enterotypes changing relative importance during the pig production cycle, confirming significant developments to be occurring in the gut microbiome within a cohort of pigs. In agreement with previous studies [7, 54], the phyla *Proteobacteria* (e.g., *E. coli/Shigella*), *Bacteroidetes*, and *Fusobacteria* were more abundant in the gut of newborn piglets. Bacteria such as *Bacteroides* are involved in degrading milk glycans [55] and regulation of intestinal immunity in early life. In addition, several genera of aerotolerant or facultative anaerobes, including *Fusobacteria*, *Clostridium*, *E. coli*/*Shigella*, and *Actinobacillus* appeared within 2 days after birth; these were collectively defined as Enterotype 1. These genera include species that considered potential pathogens in pigs, especially *E. coli* (phylum *Proteobacteria*), which is associated with enteric infections in neonatal and weaned piglets. Similarly, *Fusobacterium*, is considered as an opportunistic pathogen that can cause enteritis in piglets [56] and the genus *Actinobacillus* includes species that cause respiratory infections in pigs [57]; of interest, both genera were more abundant in 2 day old piglets than in pigs from other production stages. The results suggest that potential pathogens present in the piglet’s gut [58] could have impact on the health of the pigs in the herd. As the pigs aged, the relative abundances of these bacteria drastically decreased, with a shift to Enterotype 2, which was characterized by *Rikenellaceace* RC9 gut group*, Ruminococcaeae* UCG002, and *Bacteroides* at 22 days of age; indicating that the relative abundance of potential pathogens may naturally decrease with the maturation of the pig gut microbiome. This shifting enterotype also was characterized by an increase in Alpha diversity at 22 days of age, which is consistent with previous studies [47]. At five days post-weaning, the microbiome of pigs shifted into Enterotype 3, which was dominated by *Prevotella*, *Streptococcus*, and *Lactobacillus* genera. It has been shown that *Prevotella* is well adapted to the plant-based diet of growing pigs [59]. However, even 2 weeks after this dietary change (i.e., at day 40 of age), the pigs’ microbiome remained similar with respect to enterotypes. The microbiome in piglets shifted to the *Megasphaera-, Prevotella-, and Streptococcus-*dominated Enterotype 4 at day 54, and remained the same until day 93 (i.e., 2 weeks after moving to the finisher barn). At day 128–179 of age, the majority of pigs shifted to Enterotype 5 (dominated by *Streptococcus, Clostridium,* and *Muribaculaceae).* Similar age-specific enterotypes at each time point up to 3 years of age have been reported in humans [60]. Thus, the continuous change in microbiome composition and consistent shift of enterotypes in production-aging pigs could explain the age-related shifts among underlying gut microbial populations [61] and any associated resistome changes.

### Diversity and quantities of ARGs declined with age

Based on the shotgun metagenomic approach, all fecal samples analyzed contained 260 ARGs (i.e., gene groups) conferring resistance to 29 antimicrobial classes and by 77 known mechanisms; of these, tetracycline resistance genes, *tet*(W), *tet*(O), *tet*(40) and *tet*(Q), were the most abundant. These tetracycline resistance genes have also been reported at the highest relative abundance within the gut microbiome of antibiotic-naïve pigs [62], though such findings could partially be explained by the long-term historical use of tetracyclines in pig production system [63].

Interestingly, even though the 2-day-old piglets did not receive any antimicrobials, and the fecal microbiome was at its lowest diversity, we detected a high number of unique ARGs in their fecal microbiome; importantly, many of these confer resistance to metals, multi-compounds, and biocides (e.g., *acr*B, *acr*F, *cop*A, *cus*A, *mdt*B, mdtC *mdt*F, *znt*A). The sources and development of these metal genes and biocide resistant genes in the gut of newborn piglets remains largely unknown, but studies have shown bacteria with metal resistance gain competitive advantages over other microbes [64]. In addition, the richness and abundance of ARGs conferring resistance to metals, multi-compounds, biocides, and several other drug classes, declined as the pigs aged. Nearly 135 ARGs were detected only in the feces of pigs from 2 to 22 days of age and were not detected at any other age points. The per sample sequencing depth (raw reads generated from metagenomic sequencing) was similar across all age points, so any variations in sequencing depth should not have influenced overall findings in this regard.

We found high abundances of genes that encode for tetracycline resistance MFS efflux pumps, drug and biocide RND efflux pumps, and multi-metal resistance regulator mechanisms in feces of 2-day-old piglets. The *post-hoc* co-occurrence network analysis suggested important correlations exist among ARG type (i.e., drugs, metals, multi-compounds, and biocides) in pigs, further suggesting that antibiotic and metal/biocide resistance may co-occur [15, 65, 66]. It is possible that heavy metals, multi-compounds, and antibacterial biocides, along with drug resistance genes potentially present in gut microbiomes of newborn piglets may serve to maintain the high prevalence of AMR, even in the absence of antimicrobial selection pressure. However, further investigation is needed to identify the sources and selective pressures of these diverse ARGs, including metals and multi-compound/biocides present within the gut microbiome of the newborn piglets, because these chemicals are typically not included in routine standard AMR phenotypic assays.

Interestingly, pig feces exhibited an overall reduction in abundances of targeted ARGs, *tet*(A) and *bla*_CTX-M_, relative to 16S rRNA, with the aging of pigs. Specifically, the abundance of *tet*(A) gene showed a sharp drop between Day 22 and 26 of age; while the abundance of *bla*_CTX-M_ declined more gradually with pig’s age. The two resistant genes were targeted because the *tet*(A) gene represented a high abundant ARG of clinical importance among coliform bacteria [16], while *bla*_CTX-M_ (3rd generation cephalosporin drug class) represented a low abundance, yet critically important AMR gene within the fecal metagenomes. The declining trend in abundance of both AMR genes could readily be attributed to changes in the fecal microbial population with age, especially as both are commonly found in coliforms, which decrease with age, from piglet through market age [45]. When we evaluated quantities of the 16S rRNA gene copies (log_10_ transformed 16S rRNA gene copies measured by qPCR) as a proxy of overall of bacterial load (i.e., absolute abundance), we found that changes in total bacterial abundance across age points were similar. These results further indicate that changes or shifts in bacterial taxa within fecal metagenomes are more likely to be with age as opposed to any significant reduction in overall bacterial community DNA counts.

An age-related decline in phenotypic as well as genotypic (ARGs) quantity in fecal bacteria from pigs raised with or without antibiotics has been documented [11, 16, 67]. A recent study in wean-to-market pigs also showed that despite significant differences in the intensity of antibiotic exposures, resistome composition remained largely similar between those pigs that received minimal to moderate versus intensive antibiotic exposures [68]. Thus, overall reduction in the number of ARGs could well be due to age-related shifts in underlying microbial taxa. In fact, this pattern was seen in the 16S rRNA microbiome in our analysis, concomitant with the decrease in the relative abundance of several bacterial taxa; for example, sharply declining phylum *Proteobacteria,* which includes the coliform bacteria commonly known to house both *tet*(A) and *bla*_CTX-M_ (Fig. 1F). These have previously been found more likely to carry clinically important ARGs than other taxa [69–73]. In addition, several facultative or anaerobic genera, such as *Escherichia/Shigella*, *Actinobacillus*, *Enterococcus*, and *Clostridioides* that are predicted to harbor more ARGs [74], were dominant in 2-day-old piglets, and their abundance decreased as the pigs aged. Human studies have shown that the relative abundance of both *E. coli,* and the phylum *Proteobacteria* more generally, were highly correlated with the composition of the fecal resistome in infants [75]. Although we cannot confidently attribute the ARGs to any particular bacteria in the feces of piglets, based on our study design and methods employed, the shift of enterotypes in a cohort of pigs suggests the possible association between age-related change in microbiome composition and the associated fecal resistome dynamics.

### Counts of coliforms and enterococci with reduced antimicrobial susceptibility were greater at initial age-points.

Despite several limitations, culture-based methods have repeatedly proven to be important tools for the characterization of AMR in commensal bacteria [76]. When we performed phenotypic AMR assessments of fecal bacteria, we found that 2-day-old piglets had the highest prevalence and density of AMR coliforms and enterococci, which declined with age throughout the production cycle. At 2 days of age, phenotypic resistance in coliforms was detected in 72% of samples tested using antimicrobials (aminopenicillins, 3rd generation cephalosporins, tetracyclines, macrolides, aminoglycosides, sulfonamides, and phenicols). Further, phenotypic resistance among enterococci was detected in 63% of samples tested using antimicrobials (penicillins, quinolones, tetracyclines, macrolides, aminoglycosides, lincosamides, and nitrofuran), with the prevalence in the cohort subsequently declining for both bacterial types with increasing age of pigs. Other investigators [8, 9, 77–80] have also observed and reported higher prevalence of AMR among fecal bacteria in younger animals than in older, often adult, animals. Further, recent study reported minimal differences in phenotypic resistance of *E. coli* during the nursery-market production stages despite different intensities of antimicrobial exposure [81].

Higher numbers of ARGs, gene copies per 16S rRNA gene, and the abundance of AMR coliform or enterococci in the feces of newborn piglets at first seems counter-intuitive, especially when attempting to link the occurrence to previous or current antibiotic exposure. In the present study, all of the pigs were antibiotic naïve and thus did not receive any antimicrobials during their whole production life span. This does not preclude the potential influence of prior antimicrobial use in the same facilities for earlier production cohorts; indeed, it is well recognized that the ambient pen environment provides much of the enteric flora to the neonatal, nursery, or growing pig. Factors other than antimicrobial exposure have been shown to be associated with AMR gene levels in the feces of pigs [82]. Additionally, events such as viral illness and physical grouping of pigs have been shown to influence the dynamics of the microbiome and resistome in pigs [68]. These findings, along with our own results, suggest that factors driving AMR require investigation beyond merely focusing on antimicrobial use [83].

We speculate that the decline in carriage of resistant coliforms and enterococci with the aging of pigs may be related to changes in competitive advantage and colonization ability in the gut environment [84]. Studies have shown that multidrug-resistant *E. coli* can exhibit higher fitness compared to susceptible *E. coli* within the gut microbial community of very young animals [85], and thus are able to rapidly colonize in the naïve gut environment of the neonate [13, 79]. Extensive research on human infants has shown multiple ARGs and higher resistant bacterial populations in the infant GI tract within the first week of age; this, among babies fed either breast milk or formula and born to mothers who had no exposure to antibiotic drugs during the last trimester of pregnancy [76]. We suspect that in the low microbial diversity typical of the GI tract of newborn piglets, resistant bacteria have a greater ability to compete and are able to colonize successfully; however, as the microbial diversity increases with age, the resistant bacterial population loses its competitive advantage and thus gradually declines over time. This can only be seen as a welcome outcome, given the potential risks associated with passing AMR bacteria via the food supply, most typically at slaughter age.

Piglets experience a number of farm-related changes during a relatively short period of time, including exposure to different environments, changing of housing and associated dietary changes, all of which may influence the overall microbiome and AMR [10, 86]. However, we have considered these changes by collecting samples at nine different age points that also corresponded to periods before and after moving piglets into each new facility, as well as times when pigs received a different (e.g., nursery or finisher) diet (Fig. 1). However, the decline in ARGs and counts of resistant bacteria and gene copy numbers do not appear to be due solely to housing and associated diet changes. If these changes were mainly due to the diet, then we would have observed a greater impact, especially shortly after weaning on Days 26 and 40; however, we also observed a decline in AMR fecal bacteria associated with age, even when diet was not changed. This finding is also supported by previous studies in dairy calves [12] and pigs [84]. Further, we found the proportion of total coliform and enterococci counts were largely unchanged during the entire study period, and that AMR coliforms and enterococci declined more rapidly with the age of pigs. These findings are relative to within specific genera. Thus, these are not directly associated with major shifts in bacterial taxa. This further suggests that decline in the counts of AMR fecal bacteria were not artifactual, but reflect an actual decline due to as-yet unknown age-related phenomenon. In vivo challenge studies using strains isolated from different age groups and attempts to colonize other age groups may well show adaptations that restrict strains to certain age/production groups.

Feces reflecting a higher bacterial richness are generally considered beneficial for overall host health [87]. Furthermore, our results showed that relative abundance of several potential pathogenic bacteria, including *E. coli*, *Fusobacteria*, decreased as pig fecal microbiome matured and increased in diversity. Additionally, higher microbiome diversity in piglets was associated with decreased ARG diversity and abundance shortly after weaning. Thus, we propose that employing potential interventions prior to weaning, and immediately post-weaning (e.g., inclusion of dietary fiber, probiotic supplements, or management practices to reduce stress, etc.) in order to increase gut microbiome diversity, would influence the gut resistome and this could lower the AMR gene burden in the gut microbiome of the pig host.

Fecal microbiome largely represents the microbes present in the distal region of the hindgut, while other gut sections contain different microbial communities compared with that of the feces [88, 89]; meanwhile, the fecal microbiome is considered an important source of ARGs for other animals in the pens and other farms [90]. While our study followed a single cohort of piglets from birth to market age in order to characterize the fecal microbiome and AMR, the study has limitations. We enrolled a single cohort of pigs (*n* = 16), of which twelve pigs completed the study. Age was found to be the strongest determinant of overall microbiome variability (R^2^); however, that part of higher R^2^ value could be due to heterogenous dispersion across age groups, particularly from 2 to 40 days of age, and thus may need to be considered when we interpret the beta-diversity of the microbial community. Similarly, measurement of resistance in indicator bacteria (e.g., coliforms and enterococci) is a widely used method in studies of AMR in enteric bacterial populations, but it is clearly a narrow approach that ignores the vast numbers of bacterial phyla in the hind gut and feces of pigs. These methods likely underestimate the true magnitude of target populations (i.e., the overall microbial community) and the underlying dynamic of AMR [16]; for instance, several metal resistance genes that we detected in piglet feces by metagenomic sequencing approaches are not included in standard phenotypic assays. Metagenomic approaches allowed us to detect diverse ARGs present in any given microbial ecology; however, it is difficult to attribute observed ARGs to any particular bacterial taxa. Further, many of these ARGs are of limited or no clinical consequence, which is the major driver of most research in this realm.

While the 16S rRNA amplicon sequencing approach is widely used to characterize microbial communities in a sample, the output of this approach provides the relative abundance for each feature/taxon in each sample rather than the absolute abundance [92]. Applying absolute quantification of microbial taxa (e.g., the 16S rRNA gene copies measured by qPCR) would be more relevant in future studies in order to determine microbial changing across study groups and age/timepoints [93, 94].

## Conclusions

The fecal microbiome of a cohort of pigs exhibited consistent changes across individual pigs and became more diverse with age, while resistome followed a different trajectory with noticeably higher richness and quantities of ARGs in the newborn piglets and overall reduction of ARGs over time. The AMR indicator fecal bacteria (coliforms and enterococci) in the pig cohorts also exhibited an age-dependent dynamics for many antimicrobial drug classes, including third-generation cephalosporins, aminopenicillins, aminoglycosides, and macrolides, with the highest abundance in early ages, followed by a decrease as the age of the pigs increased. We propose that a better understanding of AMR temporal dynamics in the gut microbiome of pigs during a full production cycle will ultimately lead to interventions that can further decrease the transmission of resistant bacteria in the food chain, both during production and at slaughter. Furthermore, our finding indicates that nonantibiotic factors (e.g., age) as important determinant of AMR and also highlights opportunity for potential interventions to increased gut microbiome diversity that would possibly lower AMR gene burden within gut microbiome of host. This may also contribute to the research of human gut microbiome due to the similarity between pig and human gastrointestinal (GI) track.

## Supplementary Information

**Additional file 1**. Quantification of ARGs in fecal samples from a of cohort of piglets.

**Additional file 2: Table S2.** Antimicrobial class and concentrations used in agar media to determine bacterial phenotypic resistance.

**Additional file 3: Table S3**. Metadata and 16S rRNA sequencing read statistics for all samples included in this study.

**Additional file 4: Fig. S1**. Alpha diversity at amplicon sequent variants (ASV) level of microbiome by pig age measured by: A) richness, and B) Shannon diversity. Non-metric multidimensional scaling (NMDS) ordinating plot based on Bray–Curtis distances illustrate variation in microbial community structures at: C) ASV level, D) Family, E) Class, and F) Phylum level by age of piglets. *R*^*2*^ represents the amount of variability explained by age.

**Additional file 5: Fig. S2.** Beta-dispersion value (distance to centroid) for each age group for microbial genera.

**Additional file 6: Fig. S3**. Bacterial profiles across age in a cohort of piglets. (A) Relative abundance of different phyla significantly decreased or else increased with age. Shaded curves show 95% confidence intervals of estimates means. B) Stacked bar representing relative abundance of families over time; families with a relative abundance less than 5% were grouped into “Family < 5%” as shown in gray color. C) Quantities of 16s rRNA copies (measured by qPCR) across age-points and sex of piglets.

**Additional file 7: Table S4.** Complete list of microbial features (phyla and genera) whose abundance were associated with age (continuous variable) of piglets determined by MaAsLin 2 (Microbiome Multivariable Associations with Linear Models) approach.

**Additional file 8: Fig. S4.** Model fit for the number of Dirichlet mixture components (K) using the Laplace approximation to the negative log model.

**Additional file 9: Fig. S5.** Number of paired end raw sequence read counts (via shotgun metagenomic sequencing) generated from each sample group by pig age.

**Additional file 10: Fig. S6.** Non-metric multidimensional scaling (NMDS) ordination of pig fecal samples based on cumulative sum scaling (CSS) normalized resistome count at ARG level.

**Additional file 11: Fig. S7.** Co-occurrence networks among antimicrobial resistance genes (ARGs) in pigs. Each node represents the ARGs colored by respective class of antimicrobials (drugs, metal or multicompound and biocides) and size of each node represented the number of connections (degree of connections). MLS = Macrolides, lincosamides, and streptogramins.

**Additional file 12: Fig. S8**. Density plots with multiple imputations for standardized *bla*_CTX-M_ gene quantity data. The blue and red lines represent the observed and imputed values, respectively, from 20 imputed data sets. The completed line (green) represents both observed and imputed values. Boxplots represent distribution of standardized log_10_ *bla*_CTX-M_ gene copies per gram of feces by age (black dot = observed and red dot = imputed).

## Data Availability

The raw sequence data generated from both amplicon and shotgun metagenomes during this study are available in the NCBI repository under BioProject PRJNA861700, PRJNA878851 respectively. Metadata for all samples included in this study are presented in Additional file 1.
