# Supplementary material for: Age influences the temporal dynamics of microbiome and antimicrobial resistance genes among fecal bacteria in a cohort of production pigs
Source: Anim Microbiome. 2023 Jan 10;5:2. doi: 10.1186/s42523-022-00222-8 (PMC9830919; doi:10.1186/s42523-022-00222-8)
Supplement: Supplementary file 1 — Additional file 1. Quantification of ARGs in fecal samples from a of cohort of piglets. [file 42523_2022_222_MOESM1_ESM.docx]

**Additional File 1. Supplementary File 1**

**Quantification of AMR genes in fecal samples from a of cohort of piglets**

***Standard curve generation***

The *tet*(A) gene PCR reactions were run in a total volume of 20 μL and each reaction contained 10 μL of 2 × Brilliant III SYBR® Green QPCR Master Mix Agilent Technologies, cat # 600892), 2 μL of the positive control DNA (*E. coli* strain-*GD22,* *5043385* base pairs), 2.5 μL of each primer (10 uM), 3 μL of nuclease-free water. Similarly, 16s rRNA and *bla*_CTX-M_ gene PCR reactions were run in a total volume of 20 μl, and each reaction contained 10 μL of 2 × Brilliant III SYBR® Green QPCR Master Mix mix/low Rox, 2 μL of the positive control DNA (*E. coli* strain-GD26, 4761741 base pairs), 2.5 μL each of forward and reverse primer, and 3 μL of nuclease-free water. The positive control *E. coli* isolates (*E. coli* strain-*GD22 and E. coli* strain-*GD26*) were previous identified as harboring the *tet*(A) and *bla*CTX_-M_ gene, respectively.

**Additional File 1. Table S1**. List of primers used in quantification of AMR genes

| **Gene name** | **Primer** | **Primer Sequence** | **Reference** |
| --- | --- | --- | --- |
| *bla*_CTX-M_ | CTX-M(F) | 5′ -CGC GGT GCT GAA GAA AAG TG- 3′ |  |
|  | CTX-M(R) | 5′ -TAT CCC CCA CAA CCC AGG AA- 3′ |  |
| *tet*(A) | *tet*(A)(F) | 5′ -GCT GTT TCC TTT TGC CGG AG- 3′ |  |
|  | *tet*(A)(R) | 5′ -TGG ACA ACA TTG CTT GCA GC- 3′ |  |
| 16S rRNA | F | 5'-CCT ACG GGN GGC WGC AG- 3' | Klindworth, A., et al. (2013) |
|  | R | 5'-GAC TAC HVG GGT ATC TAA TCC-3' |  |

F - forward; R - reverse primer

Klindworth, A., Pruesse, E., Schweer, T., Peplies, J., Quast, C., Horn, M., & Glöckner, F. O. (2013). Evaluation of general 16S ribosomal RNA gene PCR primers for classical and next-generation sequencing-based diversity studies. Nucleic acids research, 41(1), e1. <https://doi.org/10.1093/nar/gks808>

***Quantification of AMR genes in samples***

The primers for *tet*(A), *bla*_CTX-M_, and 16S rRNA genes (Table S1) were used to quantify the gene copies in the unknown DNA samples with a standard curve. The DNA sample was serially diluted, and standard curves relied on 10-fold serial dilutions of 2 μL of PCR products with a known number of gene copies in 18 μL of nuclease-free water. The lowest five concentrations were used to set each standard curve. The thermal profile for the reaction for *tet*(A) genes was 95°C for 3 minutes (activation), 35 cycles of 95°C for 5 seconds, 56°C for 20 seconds and 95°C for 1 minute, 55°C for 30 seconds, and 95°C for 30 seconds. Similarly, the thermal profile for amplification of the *bla*_CTX-M_ genes was 95°C for 3 minutes, 35 cycles of 95°C for 5 seconds, 56°C for 20 seconds, 95°C for 1 minute, 55°C for 30 seconds, and 95°C for 30 seconds. The thermal profile for the 16S rRNA gene was 95°C for 5 minutes, 40 cycles of 95°C for 5 seconds, 40 cycles of 57°C for 10 seconds, 95°C for 1 minute, 55°C for 30 seconds, and 95°C for 30 seconds. After each PCR run, data were extracted using AriaMx ver. 1.0 software (Agilent, Santa Clara, CA). To examine the specificity of the applications profile, dissociation curves were run with every plate. The gene copy numbers per g of wet feces were determined by adjusting the dilution factor for each step of community DNA extraction and then standardized to the 16s rRNA gene copy numbers per gram of feces.
