# Supplementary material for: Age influences the temporal dynamics of microbiome and antimicrobial resistance genes among fecal bacteria in a cohort of production pigs
Source: Anim Microbiome. 2023 Jan 10;5:2. doi: 10.1186/s42523-022-00222-8 (PMC9830919; doi:10.1186/s42523-022-00222-8)
Supplement: Supplementary file 2 — Additional file 2: Table S2. Antimicrobial class and concentrations used in agar media to determine bacterial phenotypic resistance. [file 42523_2022_222_MOESM2_ESM.docx]

**Additional File 2: Table S2.** Antimicrobial class and concentrations used in agar media to determine bacterial phenotypic resistance

| Bacteria/media | Antimicrobial drug class | Antimicrobial drug supplemented in agar (abbreviation) | Drug concentration (µg/mL)^1^ |
| --- | --- | --- | --- |
| Coliforms / |  |  |  |
| MacConkey Agar (MAC) | Aminoglycosides | Gentamicin (GEN) | 16 |
|  |  | Streptomycin (STR) | 32 |
|  | 3^rd^ generation cephalosporins | Ceftriaxone (AXO) | 4 |
|  | Sulfonamides/folate pathway inhibitors | Sulfamethoxazole (SMX) | 512 |
|  | Macrolides | Azithromycin (AZI) | 32 |
|  | Aminopenicillins | Ampicillin (AMP) | 32 |
|  | Phenicols | Chloramphenicol (CHL) | 32 |
|  | Fluoroquinolones | Ciprofloxacin (CIP | 1 (and 0.25) |
|  |  | Enrofloxacin (ENR) | 0.125^2^ |
|  | Quinolones | Nalidixic acid (NAL) | 32 |
|  | Tetracyclines | Tetracycline (TET) | 16 |
|  |  |  |  |
| *Enterococcus* spp. |  |  |  |
| Enterococcus Agar (ENT) | Aminoglycosides | Gentamicin (GEN) | 500 |
|  |  | Streptomycin (STR) | 1024 |
|  | Lincosamides | Lincomycin (LIN) | 8 |
|  | Macrolides | Erythromycin (ERY) | 8 |
|  |  | Tylosin | 32 |
|  | Nitrofurans | Nitrofurantoin (NIT) | 128 |
|  | Penicillins | Penicillin (PEN) | 16 |
|  | Phenicols | Chloramphenicol (CHL) | 32 |
|  | Fluoroquinolones | Ciprofloxacin (CIP) | 4 |
|  |  | Enrofloxacin (ENR) | 4 |
|  | Quinolones | Nalidixic acid (NAL) | 32^3^ |
|  | Tetracyclines | Tetracycline (TET) | 16 |

^1^Breakpoints based on Clinical Laboratory Standards Institute guidelines (CLSI, 2008) or National Antimicrobial Resistance Monitoring System consensus

^2^European Committee on Antimicrobial Susceptibility Testing (epidemiological cutoff value)

^3^Adopted from *E. coli* break-point concentration
