# Supplementary material for: Age influences the temporal dynamics of microbiome and antimicrobial resistance genes among fecal bacteria in a cohort of production pigs
Source: Anim Microbiome. 2023 Jan 10;5:2. doi: 10.1186/s42523-022-00222-8 (PMC9830919; doi:10.1186/s42523-022-00222-8)
Supplement: Supplementary file 3 — Additional file 3: Table S3. Metadata and 16S rRNA sequencing read statistics for all samples included in this study. [file 42523_2022_222_MOESM3_ESM.docx]

**Additional File 3: Table S3**. Metadata and 16S rRNA sequencing read statistics for all samples included in this study

| SampleID | S. Name | Pig ID | Gender | Age  days | Housing  facility | Raw Input | From  Cutadpt | DADA2  filtered | Percentage  Input passed  filter | Denoised | Merged | Percentage of input  merged | Non-  chimeric | Percentage of input non-chimeric | Filtered  counts |
| --- | --- | --- | --- | --- | --- | --- | --- | --- | --- | --- | --- | --- | --- | --- | --- |
| VV001.A1 | VV001 | 61 | M | 2 | Farrow_wean | 86013 | 81303 | 80068 | 98.48 | 79450 | 77043 | 94.76 | 64073 | 78.81 | 64073 |
| VV002.A2 | VV002 | 64 | M | 2 | Farrow_wean | 153126 | 145704 | 143863 | 98.74 | 142808 | 136688 | 93.81 | 101667 | 69.78 | 101665 |
| VV003.A3 | VV003 | 66 | M | 2 | Farrow_wean | 149676 | 141819 | 139762 | 98.55 | 139027 | 136535 | 96.27 | 121665 | 85.79 | 121665 |
| VV004.A4 | VV004 | 67 | M | 2 | Farrow_wean | 148033 | 139712 | 136522 | 97.72 | 135265 | 128125 | 91.71 | 95328 | 68.23 | 95326 |
| VV005.A5 | VV005 | 166 | F | 2 | Farrow_wean | 137039 | 128930 | 127009 | 98.51 | 126230 | 122581 | 95.08 | 91136 | 70.69 | 91136 |
| VV006.A6 | VV006 | 169 | F | 2 | Farrow_wean | 138665 | 131425 | 129786 | 98.75 | 128858 | 122794 | 93.43 | 100756 | 76.66 | 100756 |
| VV007.A7 | VV007 | 170 | F | 2 | Farrow_wean | 147346 | 138998 | 136671 | 98.33 | 135878 | 132948 | 95.65 | 110226 | 79.3 | 110226 |
| VV008.A8 | VV008 | 171 | F | 2 | Farrow_wean | 156786 | 148617 | 146253 | 98.41 | 145107 | 138180 | 92.98 | 105230 | 70.81 | 105230 |
| VV009.A9 | VV009 | 61 | M | 22 | Farrow_wean | 117789 | 110517 | 108194 | 97.9 | 106912 | 100488 | 90.93 | 87710 | 79.36 | 87708 |
| VV010.A10 | VV010 | 64 | M | 22 | Farrow_wean | 122266 | 114565 | 112276 | 98 | 111191 | 104146 | 90.91 | 85894 | 74.97 | 85890 |
| VV011.A11 | VV011 | 66 | M | 22 | Farrow_wean | 111853 | 104810 | 102715 | 98 | 101441 | 94225 | 89.9 | 82070 | 78.3 | 82065 |
| VV012.A12 | VV012 | 67 | M | 22 | Farrow_wean | 114346 | 107023 | 104452 | 97.6 | 103411 | 95842 | 89.55 | 84013 | 78.5 | 84004 |
| VV013.B1 | VV013 | 166 | F | 22 | Farrow_wean | 92544 | 87407 | 85895 | 98.27 | 84811 | 78217 | 89.49 | 63194 | 72.3 | 63187 |
| VV014.B2 | VV014 | 169 | F | 22 | Farrow_wean | 126714 | 119417 | 115824 | 96.99 | 114414 | 108488 | 90.85 | 90353 | 75.66 | 90347 |
| VV015.B3 | VV015 | 170 | F | 22 | Farrow_wean | 133128 | 124859 | 121615 | 97.4 | 120028 | 108997 | 87.3 | 86229 | 69.06 | 86227 |
| VV016.B4 | VV016 | 171 | F | 22 | Farrow_wean | 112860 | 106376 | 104027 | 97.79 | 102523 | 94696 | 89.02 | 81232 | 76.36 | 81225 |
| VV017.B5 | VV017 | 61 | M | 26 | Nursery | 125685 | 117338 | 114253 | 97.37 | 112818 | 105101 | 89.57 | 82395 | 70.22 | 82395 |
| VV018.B6 | VV018 | 64 | M | 26 | Nursery | 117226 | 109319 | 106287 | 97.23 | 104265 | 94923 | 86.83 | 78160 | 71.5 | 78145 |
| VV019.B7 | VV019 | 66 | M | 26 | Nursery | 119961 | 110948 | 108352 | 97.66 | 106554 | 96363 | 86.85 | 77273 | 69.65 | 77271 |
| VV020.B8 | VV020 | 67 | M | 26 | Nursery | 123722 | 111995 | 109207 | 97.51 | 107801 | 100804 | 90.01 | 77709 | 69.39 | 77704 |
| VV021.B9 | VV021 | 166 | F | 26 | Nursery | 138416 | 129749 | 126122 | 97.2 | 124604 | 116445 | 89.75 | 89256 | 68.79 | 89254 |
| VV022.B10 | VV022 | 169 | F | 26 | Nursery | 126713 | 119348 | 116368 | 97.5 | 114772 | 105671 | 88.54 | 81992 | 68.7 | 81988 |
| VV023.B11 | VV023 | 170 | F | 26 | Nursery | 130153 | 122386 | 119691 | 97.8 | 118317 | 111626 | 91.21 | 79971 | 65.34 | 79963 |
| VV024.B12 | VV024 | 171 | F | 26 | Nursery | 117877 | 111576 | 109281 | 97.94 | 107717 | 98038 | 87.87 | 81017 | 72.61 | 81009 |
| VV025.C1 | VV025 | 61 | M | 40 | Nursery | 149329 | 140297 | 136474 | 97.28 | 134052 | 119995 | 85.53 | 91232 | 65.03 | 91232 |
| VV026.C2 | VV026 | 64 | M | 40 | Nursery | 144434 | 135618 | 132298 | 97.55 | 130232 | 117820 | 86.88 | 90101 | 66.44 | 90101 |
| VV027.C3 | VV027 | 66 | M | 40 | Nursery | 154418 | 145338 | 141160 | 97.13 | 138842 | 124995 | 86 | 88543 | 60.92 | 88532 |
| VV028.C4 | VV028 | 67 | M | 40 | Nursery | 145774 | 137132 | 133453 | 97.32 | 131257 | 119071 | 86.83 | 92443 | 67.41 | 92432 |
| VV029.C5 | VV029 | 166 | F | 40 | Nursery | 149546 | 140658 | 137427 | 97.7 | 135885 | 125560 | 89.27 | 91494 | 65.05 | 91481 |
| VV030.C6 | VV030 | 169 | F | 40 | Nursery | 126678 | 118463 | 115771 | 97.73 | 114577 | 107115 | 90.42 | 86351 | 72.89 | 86349 |
| VV031.C7 | VV031 | 170 | F | 40 | Nursery | 147628 | 140119 | 136762 | 97.6 | 134758 | 122293 | 87.28 | 88706 | 63.31 | 88699 |
| VV032.C8 | VV032 | 171 | F | 40 | Nursery | 152497 | 142679 | 139041 | 97.45 | 137561 | 127194 | 89.15 | 95861 | 67.19 | 95855 |
| VV033.C9 | VV033 | 61 | M | 54 | Nursery | 159728 | 148996 | 144596 | 97.05 | 142627 | 131796 | 88.46 | 97188 | 65.23 | 97181 |
| VV034.C10 | VV034 | 64 | M | 54 | Nursery | 137817 | 130106 | 126967 | 97.59 | 125150 | 114715 | 88.17 | 85485 | 65.7 | 85485 |
| VV035.C11 | VV035 | 66 | M | 54 | Nursery | 132833 | 125166 | 122494 | 97.87 | 120749 | 108943 | 87.04 | 87300 | 69.75 | 87298 |
| VV036.C12 | VV036 | 67 | M | 54 | Nursery | 105119 | 98839 | 96726 | 97.86 | 95665 | 88524 | 89.56 | 71378 | 72.22 | 71375 |
| VV037.D1 | VV037 | 166 | F | 54 | Nursery | 129761 | 123082 | 120255 | 97.7 | 118686 | 108798 | 88.39 | 84134 | 68.36 | 84129 |
| VV038.D2 | VV038 | 169 | F | 54 | Nursery | 122525 | 115323 | 112536 | 97.58 | 110721 | 100726 | 87.34 | 79657 | 69.07 | 79648 |
| VV039.D3 | VV039 | 170 | F | 54 | Nursery | 137267 | 129826 | 126834 | 97.7 | 125241 | 114408 | 88.12 | 83996 | 64.7 | 83989 |
| VV040.D4 | VV040 | 171 | F | 54 | Nursery | 130664 | 122859 | 120248 | 97.87 | 118736 | 110194 | 89.69 | 85222 | 69.37 | 85219 |
| VV041.D5 | VV041 | 61 | M | 77 | Nursery | 124820 | 117126 | 114561 | 97.81 | 112640 | 100041 | 85.41 | 76598 | 65.4 | 76593 |
| VV042.D6 | VV042 | 64 | M | 77 | Nursery | 131688 | 122901 | 119036 | 96.86 | 117179 | 106051 | 86.29 | 81933 | 66.67 | 81933 |
| VV043.D7 | VV043 | 66 | M | 77 | Nursery | 126357 | 118717 | 115880 | 97.61 | 113964 | 102399 | 86.25 | 81866 | 68.96 | 81862 |
| VV044.D8 | VV044 | 67 | M | 77 | Nursery | 140268 | 132182 | 128676 | 97.35 | 126908 | 114865 | 86.9 | 85253 | 64.5 | 85247 |
| VV045.D9 | VV045 | 166 | F | 77 | Nursery | 129153 | 121240 | 117538 | 96.95 | 115560 | 104292 | 86.02 | 85604 | 70.61 | 85589 |
| VV046.D10 | VV046 | 169 | F | 77 | Nursery | 119787 | 112794 | 110121 | 97.63 | 108269 | 97375 | 86.33 | 75984 | 67.37 | 75980 |
| VV047.D11 | VV047 | 170 | F | 77 | Nursery | 113918 | 107037 | 104984 | 98.08 | 103557 | 95891 | 89.59 | 78078 | 72.94 | 78078 |
| VV048.D12 | VV048 | 171 | F | 77 | Nursery | 130082 | 122305 | 119815 | 97.96 | 118098 | 107095 | 87.56 | 80099 | 65.49 | 80097 |
| VV049.E1 | VV049 | 61 | M | 93 | Finisher | 149509 | 140745 | 137147 | 97.44 | 134416 | 118964 | 84.52 | 96173 | 68.33 | 96160 |
| VV050.E2 | VV050 | 64 | M | 93 | Finisher | 159004 | 149775 | 146519 | 97.83 | 143930 | 129688 | 86.59 | 99874 | 66.68 | 99864 |
| VV051.E3 | VV051 | 66 | M | 93 | Finisher | 139458 | 131514 | 128532 | 97.73 | 126199 | 112921 | 85.86 | 89754 | 68.25 | 89748 |
| VV052.E4 | VV052 | 67 | M | 93 | Finisher | 132644 | 125325 | 122234 | 97.53 | 120330 | 109079 | 87.04 | 84650 | 67.54 | 84640 |
| VV053.E5 | VV053 | 166 | F | 93 | Finisher | 127240 | 118737 | 115694 | 97.44 | 112716 | 96705 | 81.44 | 77155 | 64.98 | 77151 |
| VV054.E6 | VV054 | 169 | F | 93 | Finisher | 137634 | 128660 | 124891 | 97.07 | 122791 | 109979 | 85.48 | 85923 | 66.78 | 85916 |
| VV055.E7 | VV055 | 170 | F | 93 | Finisher | 168453 | 157830 | 152944 | 96.9 | 150644 | 136079 | 86.22 | 102361 | 64.86 | 102361 |
| VV056.E8 | VV056 | 171 | F | 93 | Finisher | 128159 | 120682 | 118107 | 97.87 | 116215 | 105056 | 87.05 | 84055 | 69.65 | 84053 |
| VV057.E9 | VV057 | 61 | M | 128 | Finisher | 130162 | 122071 | 119222 | 97.67 | 117037 | 102389 | 83.88 | 77606 | 63.57 | 77604 |
| VV058.E10 | VV058 | 64 | M | 128 | Finisher | 118248 | 111639 | 109205 | 97.82 | 107295 | 96288 | 86.25 | 78008 | 69.88 | 78004 |
| VV059.E11 | VV059 | 66 | M | 128 | Finisher | 124910 | 117518 | 114619 | 97.53 | 112857 | 101617 | 86.47 | 79194 | 67.39 | 79191 |
| VV060.E12 | VV060 | 67 | M | 128 | Finisher | 129307 | 121355 | 118351 | 97.52 | 115934 | 102216 | 84.23 | 82512 | 67.99 | 82501 |
| VV061.F1 | VV061 | 166 | F | 128 | Finisher | 154406 | 141752 | 137982 | 97.34 | 135151 | 119441 | 84.26 | 92156 | 65.01 | 92152 |
| VV062.F2 | VV062 | 169 | F | 128 | Finisher | 103569 | 96267 | 93839 | 97.48 | 91608 | 78549 | 81.59 | 56817 | 59.02 | 56814 |
| VV063.F3 | VV063 | 170 | F | 128 | Finisher | 115720 | 107142 | 104777 | 97.79 | 102579 | 90197 | 84.18 | 72851 | 67.99 | 72846 |
| VV064.F4 | VV064 | 171 | F | 128 | Finisher | 120673 | 111539 | 109093 | 97.81 | 106970 | 93206 | 83.56 | 75675 | 67.85 | 75673 |
| VV065.F5 | VV065 | 61 | M | 179 | Finisher | 126925 | 116123 | 112721 | 97.07 | 109935 | 93605 | 80.61 | 72893 | 62.77 | 72882 |
| VV066.F6 | VV066 | 64 | M | 179 | Finisher | 127621 | 117340 | 113969 | 97.13 | 111431 | 96315 | 82.08 | 79993 | 68.17 | 79985 |
| VV067.F7 | VV067 | 66 | M | 179 | Finisher | 123200 | 113704 | 110266 | 96.98 | 107794 | 95164 | 83.69 | 80297 | 70.62 | 80285 |
| VV068.F8 | VV068 | 67 | M | 179 | Finisher | 123557 | 114314 | 112006 | 97.98 | 109720 | 95854 | 83.85 | 79770 | 69.78 | 79764 |
| VV069.F9 | VV069 | 166 | F | 179 | Finisher | 136802 | 125640 | 122384 | 97.41 | 119933 | 105627 | 84.07 | 82468 | 65.64 | 82455 |
| VV070.F10 | VV070 | 169 | F | 179 | Finisher | 113991 | 104624 | 102115 | 97.6 | 99436 | 84531 | 80.8 | 68537 | 65.51 | 68535 |
| VV071.F11 | VV071 | 170 | F | 179 | Finisher | 116398 | 107165 | 104457 | 97.47 | 102214 | 87898 | 82.02 | 72393 | 67.55 | 72390 |
| VV072.F12 | VV072 | 171 | F | 179 | Finisher | 126395 | 116573 | 113955 | 97.75 | 111281 | 94697 | 81.23 | 76259 | 65.42 | 76259 |
