# Supplementary figures and images for: Age influences the temporal dynamics of microbiome and antimicrobial resistance genes among fecal bacteria in a cohort of production pigs
Source: Anim Microbiome. 2023 Jan 10;5:2. doi: 10.1186/s42523-022-00222-8 (PMC9830919; doi:10.1186/s42523-022-00222-8)

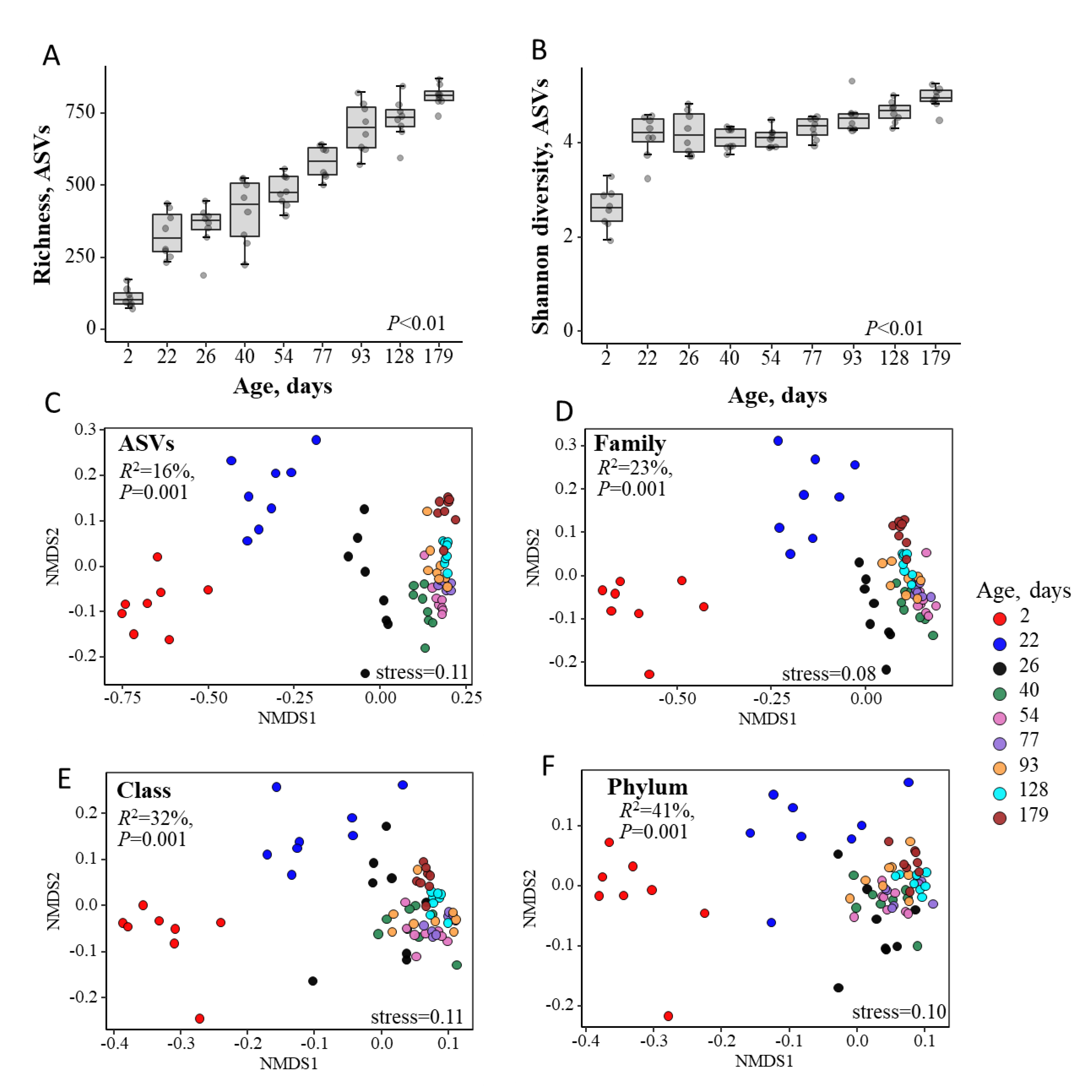

Supplement: Supplementary file 4 — Additional file 4: Fig. S1. Alpha diversity at amplicon sequent variants (ASV) level of microbiome by pig age measured by: A) richness, and B) Shannon diversity. Non-metric multidimensional scaling (NMDS) ordinating plot based on Bray–Curtis distances illustrate variation in microbial community structures at: C) ASV level, D) Family, E) Class, and F) Phylum level by age of piglets. R2 represents the amount of variability explained by age. [file 42523_2022_222_MOESM4_ESM.tif]

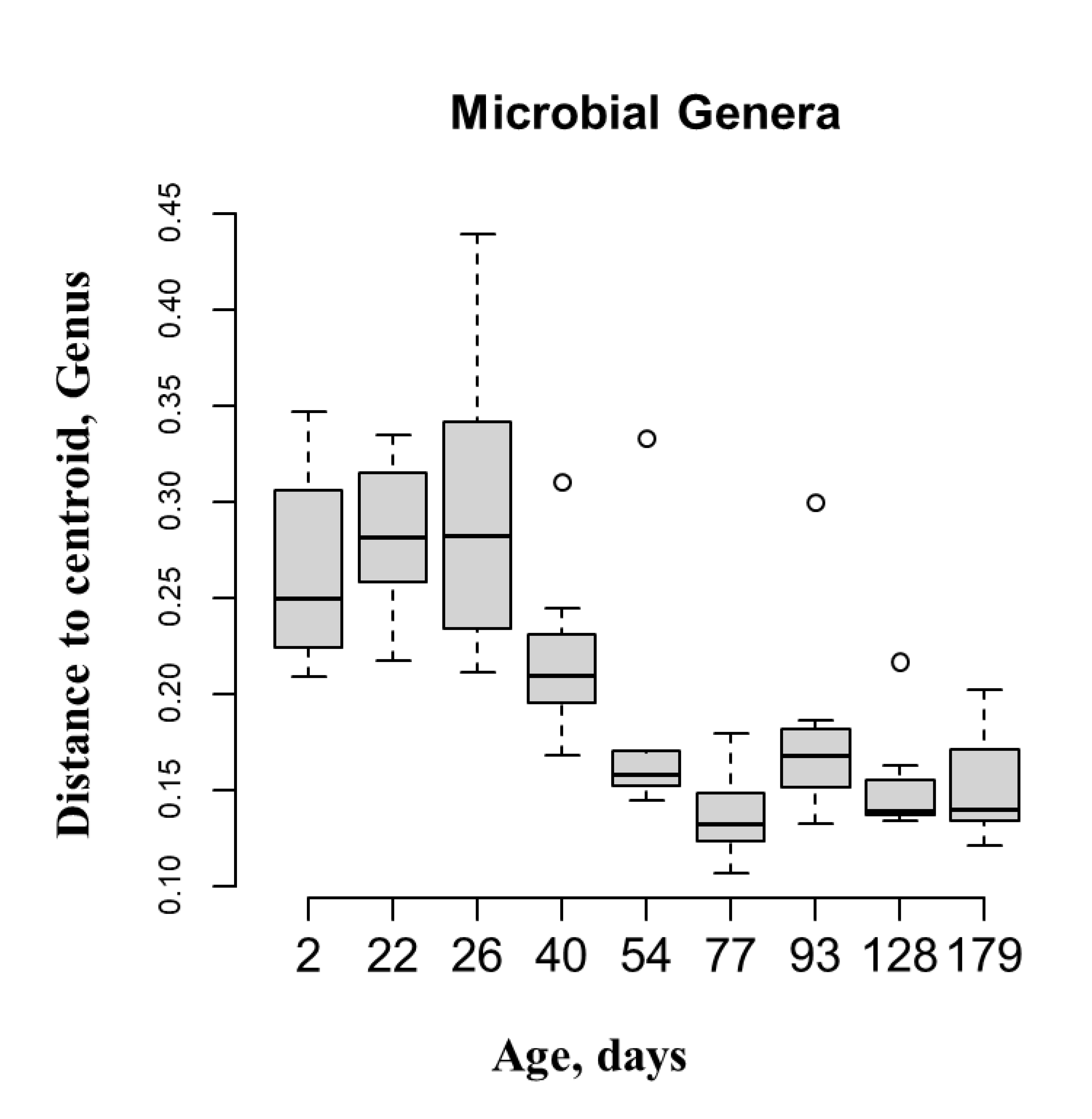

Supplement: Supplementary file 5 — Additional file 5: Fig. S2. Beta-dispersion value (distance to centroid) for each age group for microbial genera. [file 42523_2022_222_MOESM5_ESM.tif]

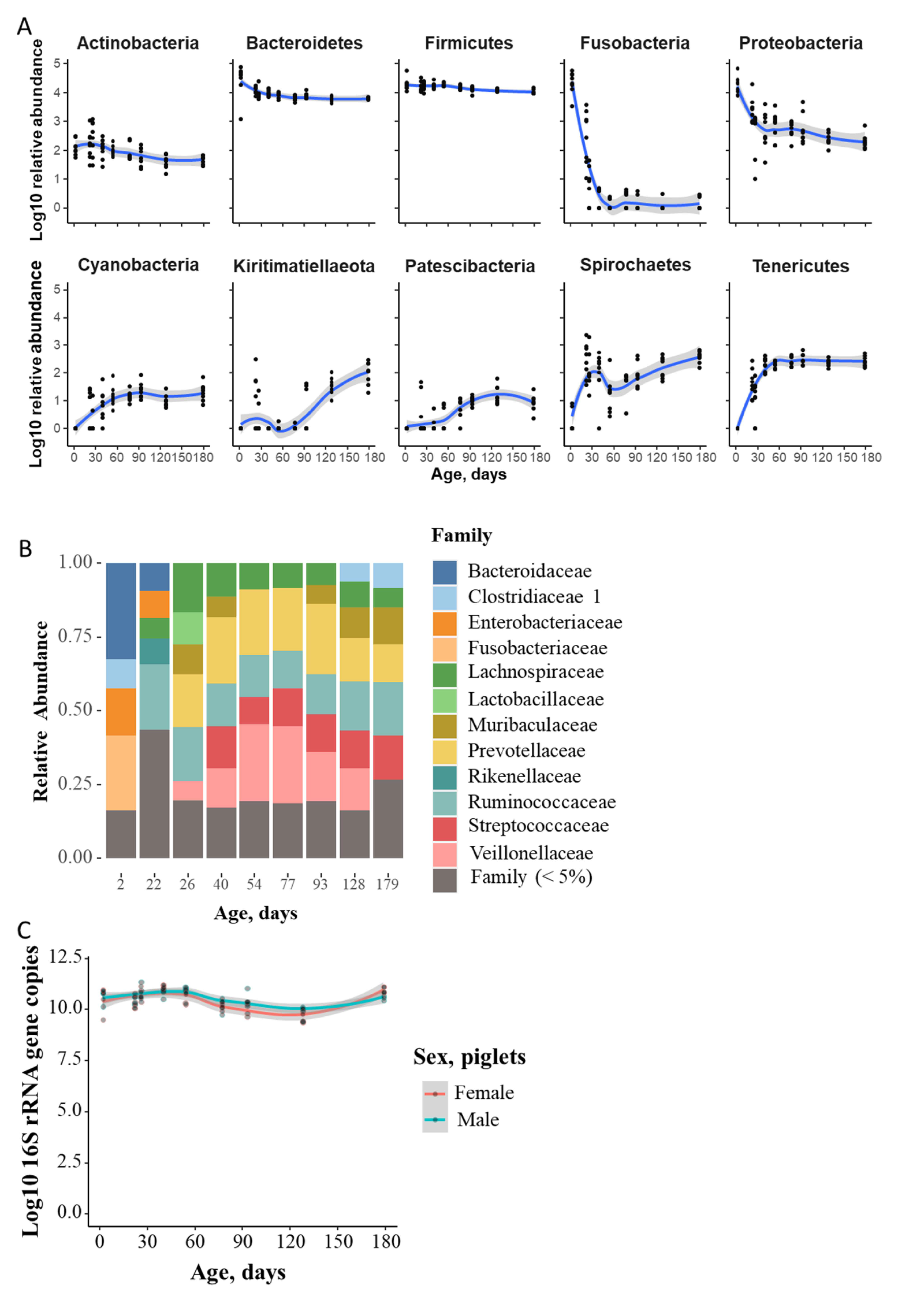

Supplement: Supplementary file 6 — Additional file 6: Fig. S3. Bacterial profiles across age in a cohort of piglets. (A) Relative abundance of different phyla significantly decreased or else increased with age. Shaded curves show 95% confidence intervals of estimates means. B) Stacked bar representing relative abundance of families over time; families with a relative abundance less than 5% were grouped into “Family < 5%” as shown in gray color. C) Quantities of 16s rRNA copies (measured by qPCR) across age-points and sex of piglets. [file 42523_2022_222_MOESM6_ESM.tif]

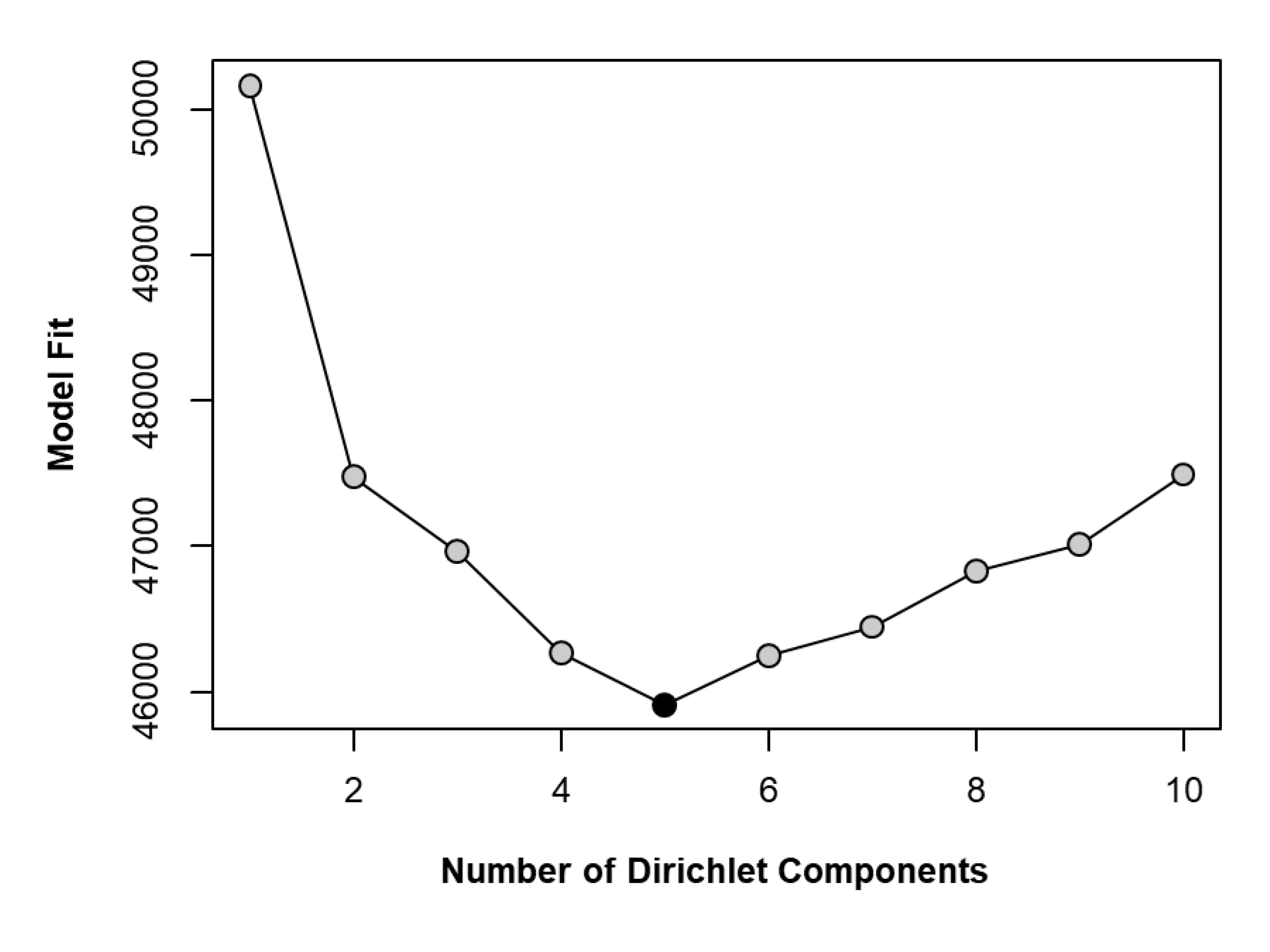

Supplement: Supplementary file 8 — Additional file 8: Fig. S4. Model fit for the number of Dirichlet mixture components (K) using the Laplace approximation to the negative log model. [file 42523_2022_222_MOESM8_ESM.tif]

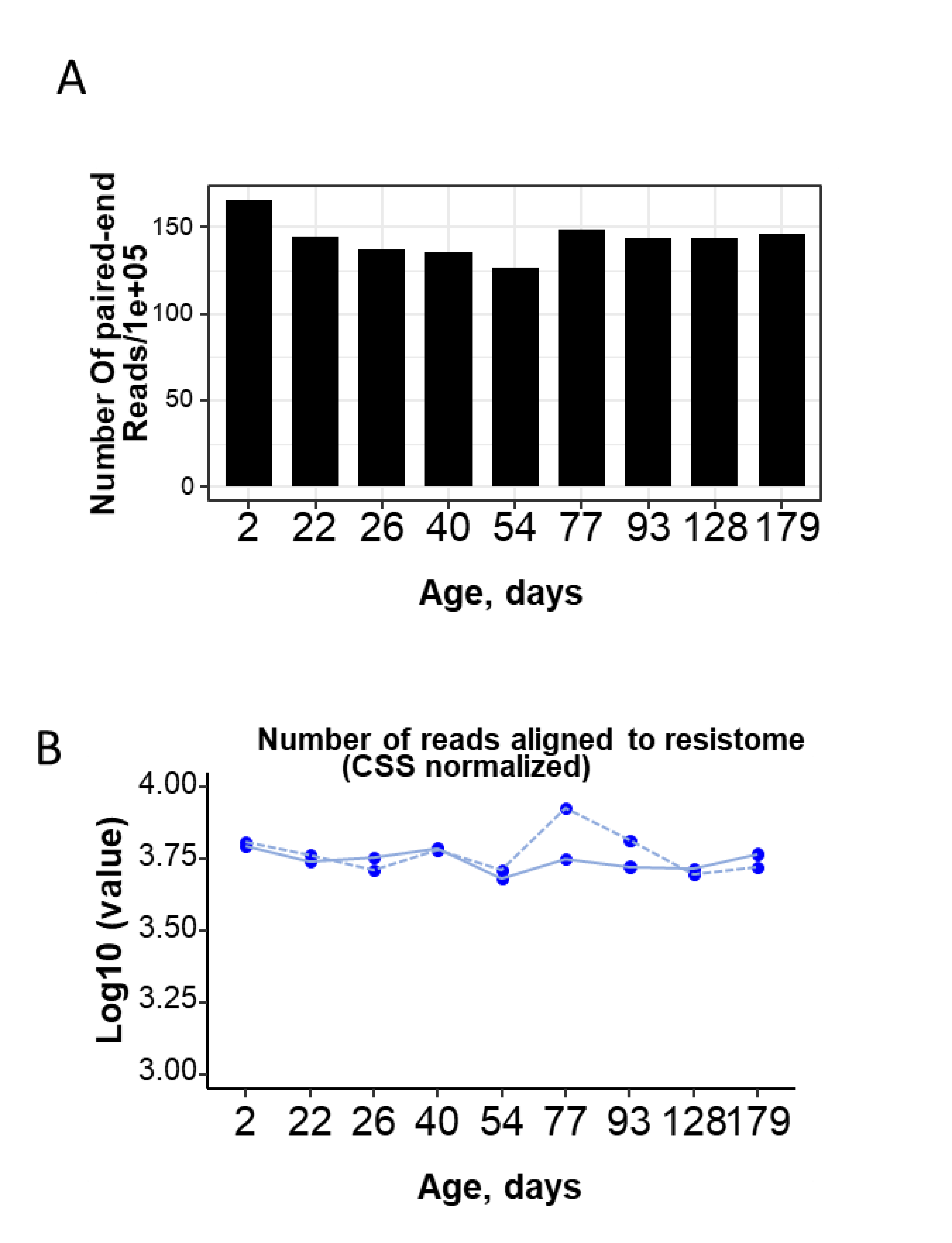

Supplement: Supplementary file 9 — Additional file 9: Fig. S5. Number of paired end raw sequence read counts (via shotgun metagenomic sequencing) generated from each sample group by pig age. [file 42523_2022_222_MOESM9_ESM.tif]

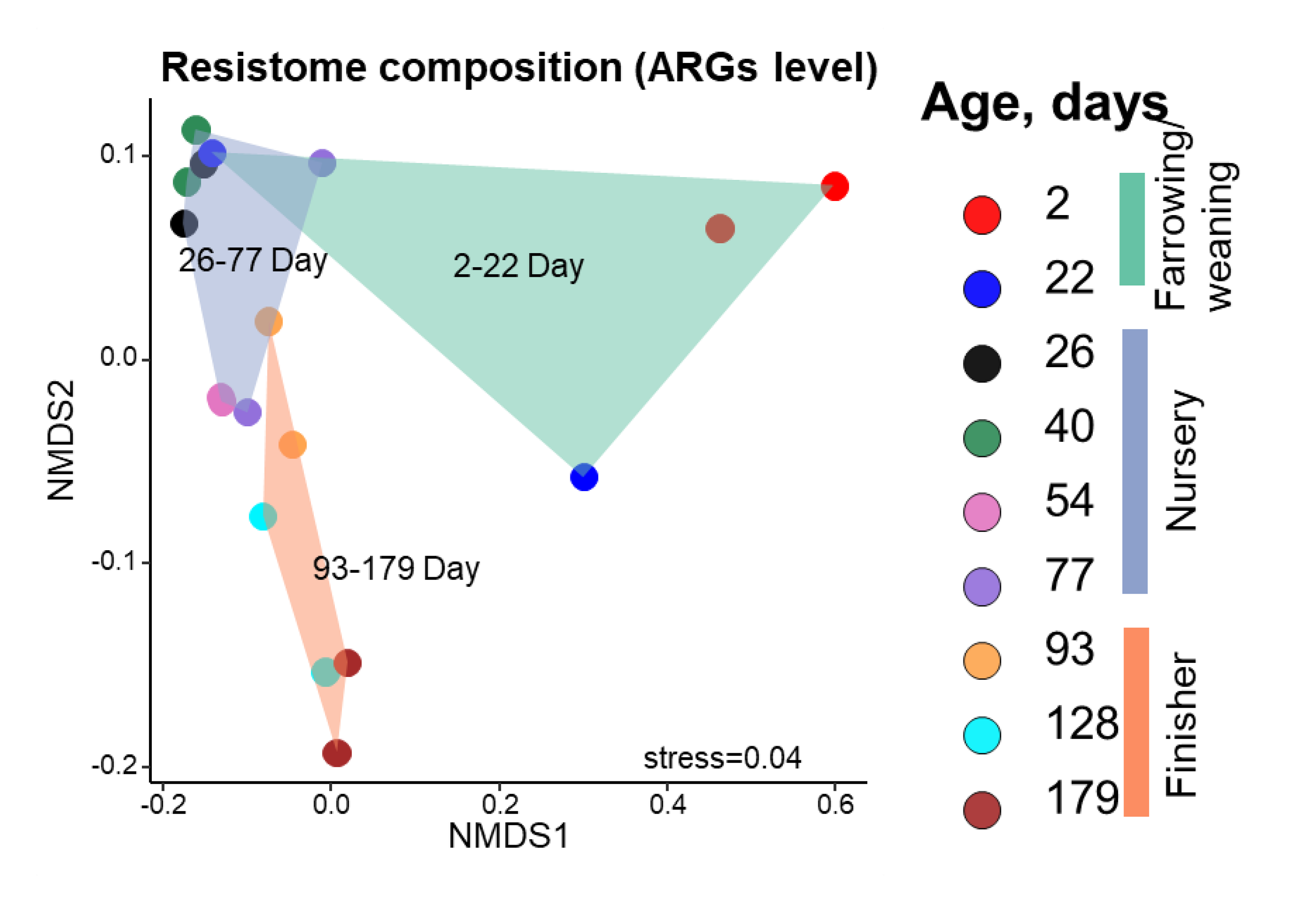

Supplement: Supplementary file 10 — Additional file 10: Fig. S6. Non-metric multidimensional scaling (NMDS) ordination of pig fecal samples based on cumulative sum scaling (CSS) normalized resistome count at ARG level. [file 42523_2022_222_MOESM10_ESM.tif]

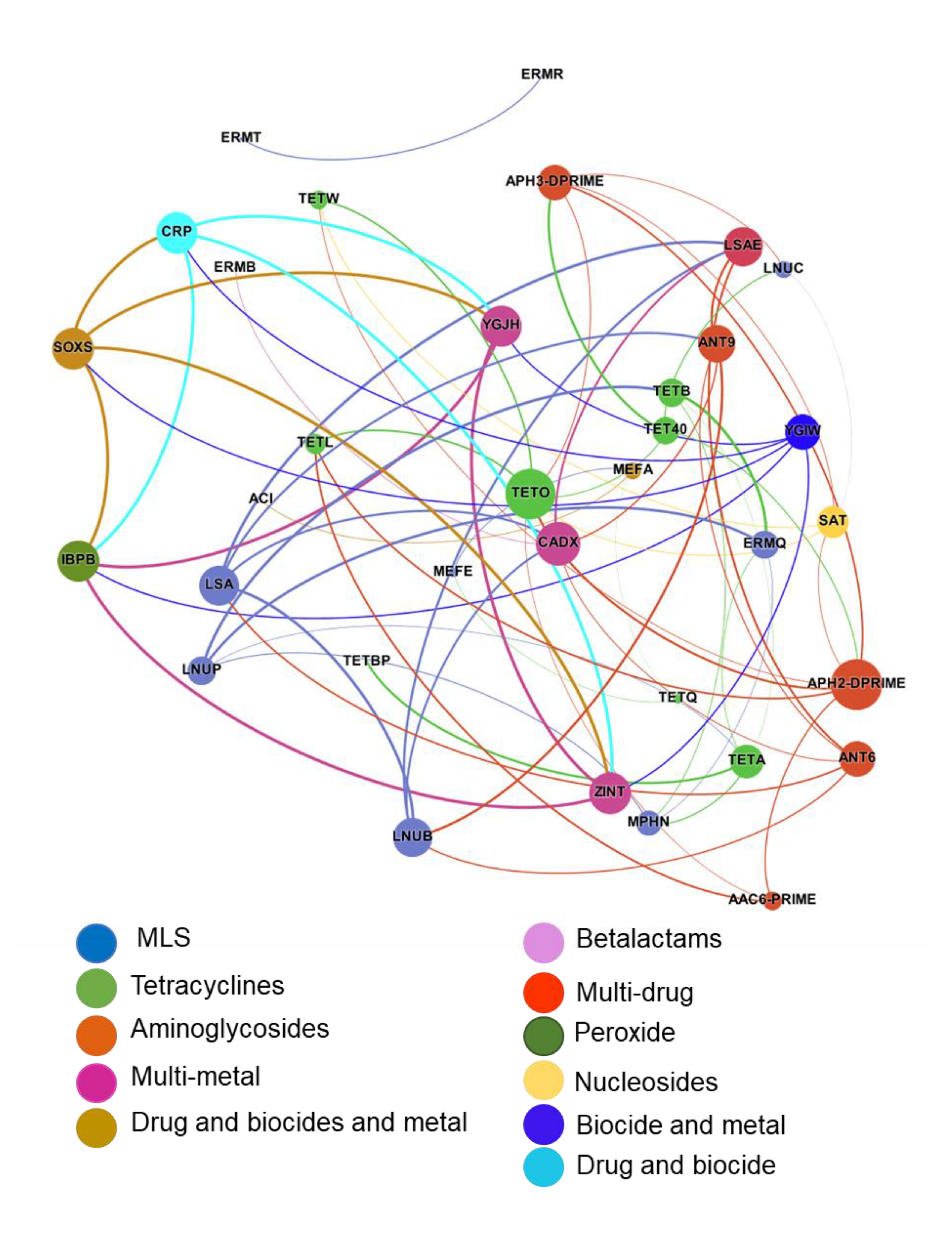

Supplement: Supplementary file 11 — Additional file 11: Fig. S7. Co-occurrence networks among antimicrobial resistance genes (ARGs) in pigs. Each node represents the ARGs colored by respective class of antimicrobials (drugs, metal or multicompound and biocides) and size of each node represented the number of connections (degree of connections). MLS = Macrolides, lincosamides, and streptogramins. [file 42523_2022_222_MOESM11_ESM.tif]

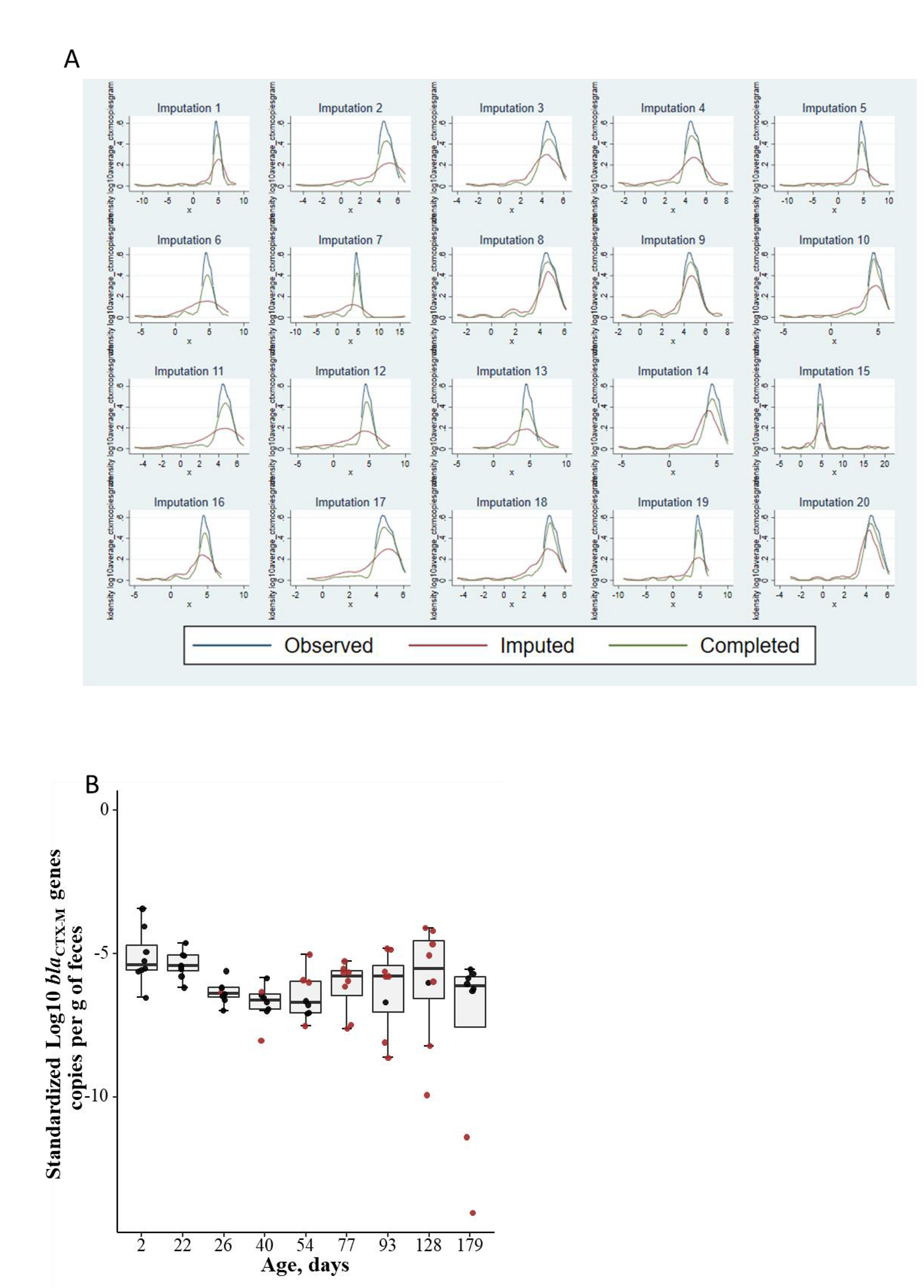

Supplement: Supplementary file 12 — Additional file 12: Fig. S8. Density plots with multiple imputations for standardized blaCTX-M gene quantity data. The blue and red lines represent the observed and imputed values, respectively, from 20 imputed data sets. The completed line (green) represents both observed and imputed values. Boxplots represent distribution of standardized log10 blaCTX-M gene copies per gram of feces by age (black dot = observed and red dot = imputed). [file 42523_2022_222_MOESM12_ESM.tif]
