# Supplementary material for: Age influences the temporal dynamics of microbiome and antimicrobial resistance genes among fecal bacteria in a cohort of production pigs
Source: Anim Microbiome. 2023 Jan 10;5:2. doi: 10.1186/s42523-022-00222-8 (PMC9830919; doi:10.1186/s42523-022-00222-8)
Supplement: Supplementary file 7 — Additional file 7: Table S4. Complete list of microbial features (phyla and genera) whose abundance were associated with age (continuous variable) of piglets determined by MaAsLin 2 (Microbiome Multivariable Associations with Linear Models) approach. [file 42523_2022_222_MOESM7_ESM.docx]

**Additional File 7: Table S4.** Complete list of microbial features (phyla and genera) whose relative abundance were associated with age (continuous variable) of piglets determined by MaAsLin 2 (Microbiome Multivariable Associations with Linear Models) approach.

| **Feature** | **Taxa**  **level** | **Coefficient** | **Standard error** | **N** | **N not zero** | **P-value** | **Q- value** | **Increased abundance with age? [1=Yes, 0=No]** |
| --- | --- | --- | --- | --- | --- | --- | --- | --- |
| Fusobacteria | Phylum | -0.01647 | 0.002594 | 72 | 39 | 1.95E-08 | 1.36E-07 | 0 |
| Proteobacteria | Phylum | -0.00732 | 0.001304 | 72 | 72 | 3.89E-07 | 1.89E-06 | 0 |
| Actinobacteria | Phylum | -0.00371 | 0.000766 | 72 | 72 | 8.74E-06 | 3.30E-05 | 0 |
| Bacteroidetes | Phylum | -0.00261 | 0.000528 | 72 | 72 | 5.18E-06 | 2.20E-05 | 0 |
| Firmicutes | Phylum | -0.00154 | 0.000254 | 72 | 72 | 6.21E-08 | 3.52E-07 | 0 |
| Patescibacteria | Phylum | 0.00674 | 0.000922 | 72 | 39 | 3.58E-10 | 1.22E-08 | 1 |
| Spirochaetes | Phylum | 0.006791 | 0.001677 | 72 | 65 | 0.000132 | 0.00045 | 1 |
| Cyanobacteria | Phylum | 0.007078 | 0.001102 | 72 | 56 | 2.00E-08 | 1.36E-07 | 1 |
| Kiritimatiellaeota | Phylum | 0.010192 | 0.00146 | 72 | 42 | 2.16E-09 | 3.68E-08 | 1 |
| Tenericutes | Phylum | 0.010623 | 0.001673 | 72 | 63 | 1.96E-08 | 1.36E-07 | 1 |
| Lachnospiraceae XPB1014 group | Genus | 0.013353 | 0.001397 | 72 | 22 | 2.92E-14 | 3.94E-12 | 1 |
| Terrisporobacter | Genus | 0.012265 | 0.001489 | 72 | 50 | 7.32E-12 | 3.30E-10 | 1 |
| Lachnospiraceae AC2044 group | Genus | 0.012175 | 0.001491 | 72 | 32 | 1.00E-11 | 3.87E-10 | 1 |
| Prevotellaceae UCG-003 | Genus | 0.011176 | 0.001679 | 72 | 63 | 5.50E-09 | 8.26E-08 | 1 |
| Turicibacter | Genus | 0.011094 | 0.001457 | 72 | 45 | 1.02E-10 | 2.50E-09 | 1 |
| dgA-11 gut group | Genus | 0.010832 | 0.001351 | 72 | 49 | 1.83E-11 | 5.50E-10 | 1 |
| Oribacterium | Genus | 0.010384 | 0.001495 | 72 | 56 | 1.66E-09 | 2.98E-08 | 1 |
| Anaerovibrio | Genus | 0.010336 | 0.001784 | 72 | 57 | 1.87E-07 | 1.80E-06 | 1 |
| Ruminococcaceae UCG-014 | Genus | 0.009425 | 0.001931 | 72 | 62 | 6.56E-06 | 3.94E-05 | 1 |
| Prevotellaceae UCG-004 | Genus | 0.009345 | 0.001233 | 72 | 47 | 1.17E-10 | 2.64E-09 | 1 |
| Prevotella 9 | Genus | 0.009328 | 0.002459 | 72 | 63 | 0.000315 | 0.001393 | 1 |
| Ruminococcaceae NK4A214 group | Genus | 0.009015 | 0.001581 | 72 | 62 | 2.70E-07 | 2.51E-06 | 1 |
| Oscillospira | Genus | 0.008892 | 0.001474 | 72 | 62 | 7.09E-08 | 7.36E-07 | 1 |
| Ruminococcaceae UCG-010 | Genus | 0.008888 | 0.001399 | 72 | 63 | 1.94E-08 | 2.50E-07 | 1 |
| Prevotella 1 | Genus | 0.008788 | 0.002067 | 72 | 57 | 6.54E-05 | 0.000321 | 1 |
| Coprococcus 1 | Genus | 0.008782 | 0.00115 | 72 | 52 | 9.28E-11 | 2.50E-09 | 1 |
| Lachnospira | Genus | 0.008671 | 0.001307 | 72 | 48 | 6.03E-09 | 8.58E-08 | 1 |
| uncultured prokaryote | Genus | 0.008604 | 0.000798 | 72 | 26 | 1.95E-16 | 5.25E-14 | 1 |
| Ruminococcaceae UCG-005 | Genus | 0.008575 | 0.001518 | 72 | 63 | 3.32E-07 | 2.96E-06 | 1 |
| Prevotella 7 | Genus | 0.008554 | 0.002315 | 72 | 49 | 0.000437 | 0.00185 | 1 |
| Treponema 2 | Genus | 0.008496 | 0.001733 | 72 | 62 | 6.03E-06 | 3.79E-05 | 1 |
| Lachnospiraceae NK4A136 group | Genus | 0.008375 | 0.001714 | 72 | 56 | 6.40E-06 | 3.93E-05 | 1 |
| [Eubacterium] ruminantium group | Genus | 0.008326 | 0.001316 | 72 | 47 | 2.17E-08 | 2.66E-07 | 1 |
| Ruminococcaceae UCG-009 | Genus | 0.008224 | 0.000977 | 72 | 49 | 3.46E-12 | 1.87E-10 | 1 |
| Oscillibacter | Genus | 0.007964 | 0.001279 | 72 | 58 | 3.22E-08 | 3.62E-07 | 1 |
| Anaeroplasma | Genus | 0.007932 | 0.000885 | 72 | 33 | 3.49E-13 | 3.14E-11 | 1 |
| unidentified rumen bacterium RF39 | Genus | 0.007921 | 0.000919 | 72 | 30 | 3.02E-12 | 1.87E-10 | 1 |
| Ruminococcaceae UCG-013 | Genus | 0.007866 | 0.001166 | 72 | 63 | 3.86E-09 | 6.13E-08 | 1 |
| Prevotellaceae UCG-001 | Genus | 0.007819 | 0.000945 | 72 | 32 | 1.19E-11 | 4.02E-10 | 1 |
| Mitsuokella | Genus | 0.007802 | 0.001802 | 72 | 54 | 4.96E-05 | 0.000248 | 1 |
| Candidatus Soleaferrea | Genus | 0.007672 | 0.001213 | 72 | 53 | 2.97E-08 | 3.48E-07 | 1 |
| Prevotellaceae NK3B31 group | Genus | 0.007665 | 0.001768 | 72 | 64 | 4.84E-05 | 0.000246 | 1 |
| Intestinibacter | Genus | 0.007617 | 0.001272 | 72 | 52 | 8.53E-08 | 8.53E-07 | 1 |
| Ruminococcaceae UCG-002 | Genus | 0.007446 | 0.001974 | 72 | 64 | 0.000337 | 0.001469 | 1 |
| Ruminococcus 1 | Genus | 0.007158 | 0.001603 | 72 | 58 | 3.03E-05 | 0.000167 | 1 |
| Candidatus Saccharimonas | Genus | 0.006875 | 0.000917 | 72 | 38 | 2.69E-10 | 5.59E-09 | 1 |
| Roseburia | Genus | 0.006862 | 0.001521 | 72 | 62 | 2.57E-05 | 0.000144 | 1 |
| Succinivibrio | Genus | 0.0068 | 0.002114 | 72 | 55 | 0.001978 | 0.007121 | 1 |
| [Anaerorhabdus] furcosa group | Genus | 0.006552 | 0.000965 | 72 | 46 | 3.15E-09 | 5.32E-08 | 1 |
| Subdoligranulum | Genus | 0.006533 | 0.001868 | 72 | 63 | 0.000826 | 0.003281 | 1 |
| Shuttleworthia | Genus | 0.006517 | 0.001273 | 72 | 34 | 2.62E-06 | 1.74E-05 | 1 |
| Family XIII UCG-001 | Genus | 0.006363 | 0.000908 | 72 | 58 | 1.30E-09 | 2.51E-08 | 1 |
| Streptococcus | Genus | 0.006348 | 0.001428 | 72 | 72 | 3.29E-05 | 0.000177 | 1 |
| Erysipelotrichaceae UCG-004 | Genus | 0.006246 | 0.001096 | 72 | 62 | 3.40E-07 | 2.96E-06 | 1 |
| Solobacterium | Genus | 0.006216 | 0.001348 | 72 | 58 | 1.79E-05 | 0.000103 | 1 |
| Rikenellaceae RC9 gut group | Genus | 0.0059 | 0.001859 | 72 | 66 | 0.002246 | 0.007776 | 1 |
| Alloprevotella | Genus | 0.005882 | 0.001703 | 72 | 67 | 0.000951 | 0.003723 | 1 |
| uncultured Porphyromonadaceae bacterium | Genus | 0.005863 | 0.001544 | 72 | 71 | 0.00031 | 0.001393 | 1 |
| Peptococcus | Genus | 0.005811 | 0.001125 | 72 | 58 | 2.64E-06 | 1.74E-05 | 1 |
| Coprococcus 2 | Genus | 0.005422 | 0.001468 | 72 | 42 | 0.000439 | 0.00185 | 1 |
| Lachnospiraceae UCG-004 | Genus | 0.005377 | 0.000884 | 72 | 48 | 5.88E-08 | 6.35E-07 | 1 |
| Christensenellaceae R-7 group | Genus | 0.005316 | 0.001881 | 72 | 63 | 0.006159 | 0.02028 | 1 |
| [Eubacterium] eligens group | Genus | 0.005247 | 0.001423 | 72 | 55 | 0.000448 | 0.00186 | 1 |
| Romboutsia | Genus | 0.004816 | 0.001831 | 72 | 34 | 0.010512 | 0.033004 | 1 |
| [Eubacterium] hallii group | Genus | 0.004766 | 0.0013 | 72 | 60 | 0.00048 | 0.001962 | 1 |
| Erysipelotrichaceae UCG-006 | Genus | 0.004748 | 0.001485 | 72 | 45 | 0.002103 | 0.007471 | 1 |
| Family XIII AD3011 group | Genus | 0.004569 | 0.001349 | 72 | 64 | 0.001176 | 0.004348 | 1 |
| Ruminiclostridium 6 | Genus | 0.004169 | 0.001097 | 72 | 53 | 0.000308 | 0.001393 | 1 |
| Ruminococcaceae UCG-008 | Genus | 0.004158 | 0.001594 | 72 | 60 | 0.011142 | 0.034186 | 1 |
| Phascolarctobacterium | Genus | 0.004131 | 0.00151 | 72 | 67 | 0.007879 | 0.025632 | 1 |
| Coprococcus 3 | Genus | 0.003962 | 0.001228 | 72 | 58 | 0.001918 | 0.006997 | 1 |
| Lachnospiraceae NK3A20 group | Genus | 0.003734 | 0.001223 | 72 | 28 | 0.003213 | 0.010843 | 1 |
| Sutterella | Genus | 0.003584 | 0.001429 | 72 | 55 | 0.014489 | 0.043468 | 1 |
| [Eubacterium] xylanophilum group | Genus | 0.003462 | 0.001322 | 72 | 48 | 0.010868 | 0.03373 | 1 |
| Lachnospiraceae NC2004 group | Genus | 0.003451 | 0.001145 | 72 | 39 | 0.003613 | 0.012042 | 1 |
| Selenomonas 1 | Genus | 0.003366 | 0.001247 | 72 | 28 | 0.008739 | 0.028089 | 1 |
| Pseudoflavonifractor | Genus | -0.00357 | 0.001408 | 72 | 11 | 0.013423 | 0.040722 | 0 |
| Cloacibacillus | Genus | -0.00445 | 0.001413 | 72 | 14 | 0.002423 | 0.008281 | 0 |
| Hydrogenoanaerobacterium | Genus | -0.00448 | 0.001308 | 72 | 13 | 0.00104 | 0.004012 | 0 |
| Ruminiclostridium 9 | Genus | -0.00456 | 0.001431 | 72 | 68 | 0.002186 | 0.007665 | 0 |
| [Ruminococcus] gnavus group | Genus | -0.0049 | 0.00144 | 72 | 8 | 0.001103 | 0.004137 | 0 |
| Bilophila | Genus | -0.00498 | 0.001373 | 72 | 16 | 0.000551 | 0.002221 | 0 |
| Alistipes | Genus | -0.0053 | 0.001969 | 72 | 35 | 0.008924 | 0.028348 | 0 |
| Butyricimonas | Genus | -0.00605 | 0.001772 | 72 | 27 | 0.001083 | 0.004118 | 0 |
| Enterococcus | Genus | -0.00617 | 0.001406 | 72 | 11 | 3.96E-05 | 0.00021 | 0 |
| [Eubacterium] fissicatena group | Genus | -0.00642 | 0.001464 | 72 | 21 | 4.09E-05 | 0.000212 | 0 |
| UBA1819 | Genus | -0.00712 | 0.001451 | 72 | 18 | 6.03E-06 | 3.79E-05 | 0 |
| Clostridioides | Genus | -0.00714 | 0.001767 | 72 | 8 | 0.000137 | 0.000648 | 0 |
| Lactobacillus | Genus | -0.00728 | 0.001298 | 72 | 71 | 4.89E-07 | 3.89E-06 | 0 |
| Clostridium sensu stricto 2 | Genus | -0.00741 | 0.001793 | 72 | 9 | 9.94E-05 | 0.000479 | 0 |
| Tyzzerella | Genus | -0.00742 | 0.001897 | 72 | 30 | 0.000213 | 0.000993 | 0 |
| Hungatella | Genus | -0.00768 | 0.001478 | 72 | 15 | 1.96E-06 | 1.36E-05 | 0 |
| Eisenbergiella | Genus | -0.00855 | 0.001613 | 72 | 27 | 1.32E-06 | 9.39E-06 | 0 |
| Intestinimonas | Genus | -0.00893 | 0.001846 | 72 | 28 | 7.70E-06 | 4.52E-05 | 0 |
| Veillonella | Genus | -0.00908 | 0.001622 | 72 | 21 | 4.04E-07 | 3.32E-06 | 0 |
| Lachnoclostridium | Genus | -0.00945 | 0.001715 | 72 | 66 | 5.74E-07 | 4.42E-06 | 0 |
| Actinobacillus | Genus | -0.01078 | 0.001961 | 72 | 24 | 6.15E-07 | 4.61E-06 | 0 |
| Escherichia-Shigella | Genus | -0.01323 | 0.002483 | 72 | 67 | 1.17E-06 | 8.57E-06 | 0 |
| Bacteroides | Genus | -0.01504 | 0.002687 | 72 | 65 | 4.06E-07 | 3.32E-06 | 0 |
| Fusobacterium | Genus | -0.01647 | 0.002594 | 72 | 39 | 1.95E-08 | 2.50E-07 | 0 |

*Coefficient*- the model coefficient value (effect size*), Standard error*: the standard error from the model; *N:* the total number of samples used in the model for this association *N not zero* the total of number of samples in which the feature was non-zero. *P-value*: the significance of this association. *Q-value:* the corrected significance was computed with *p.adjust* using the Benjamin–Hochberg correction method.
